# Genetics Responses to Hypoxia and Reoxygenation Stress in *Larimichthys crocea* Revealed via Transcriptome Analysis and Weighted Gene Co-Expression Network

**DOI:** 10.3390/ani11113021

**Published:** 2021-10-20

**Authors:** Yibo Zhang, Jie Ding, Cheng Liu, Shengyu Luo, Xinming Gao, Yuanjie Wu, Jingqian Wang, Xuelei Wang, Xiongfei Wu, Weiliang Shen, Junquan Zhu

**Affiliations:** 1 Key Laboratory of Applied Marine Biotechnology of Ministry of Education, College of Marine Sciences, Ningbo University, 169 South Qixing Road, Ningbo 315832, China; zyb15058456761@163.com (Y.Z.); dj13821799852@163.com (J.D.); 1801091036@nbu.edu.cn (C.L.); 1701091031@nbu.edu.cn (S.L.); nbugxm4851@163.com (X.G.); 1911091104@nbu.edu.cn (Y.W.); 2001130069@nbu.edu.cn (J.W.); 2 State Key Laboratory of Large Yellow Croaker Breeding, Ningbo Academy of Oceanology and Fishery, Juxian Road, Ningbo 315103, China; xlwang126@163.com (X.W.); wxiongfei@hotmail.com (X.W.)

**Keywords:** hypoxia, transcriptome, WGCNA, hub genes, *Larimichthys crocea*

## Abstract

**Simple Summary:**

Hypoxia, which occurs frequently in aquaculture, can cause serious harm to all aspects of the growth, reproduction and metabolism of cultured fish. Due to the intolerance of *Larimichthys crocea* to hypoxia, *Larimichthys crocea* often floats head or even dies under hypoxic environment. However, the molecular mechanism of hypoxia tolerance in *Larimichthys crocea* has not been fully described. Therefore, the aim of this study was to explore the hub regulatory genes under hypoxic stress environment by transcriptome analysis of three key tissues (liver, blood and gill) in *Larimichthys crocea*. We identified a number of important genes that exercise different regulatory functions. Overall, this study will provide important clues to the molecular mechanisms of hypoxia tolerance in *Larimichthys crocea*.

**Abstract:**

The large yellow croaker (*Larimichthys crocea*) is an important marine economic fish in China; however, its intolerance to hypoxia causes widespread mortality. To understand the molecular mechanisms underlying hypoxia tolerance in *L. crocea*, the transcriptome gene expression profiling of three different tissues (blood, gills, and liver) of *L. crocea* exposed to hypoxia and reoxygenation stress were performed. In parallel, the gene relationships were investigated based on weighted gene co-expression network analysis (WGCNA). Accordingly, the Gene Ontology and Kyoto Encyclopedia of Genes and Genomes enrichment analysis showed that several pathways (e.g., energy metabolism, signal transduction, oxygen transport, and osmotic regulation) may be involved in the response of *L. crocea* to hypoxia and reoxygenation stress. In addition, also, four key modules (darkorange, magenta, saddlebrown, and darkolivegreen) that were highly relevant to the samples were identified by WGCNA. Furthermore, some hub genes within the association module, including *RPS16, EDRF1, KCNK5, SNAT2, PFKL, GSK-3β,* and *PIK3CD,* were found. This is the first study to report the co-expression patterns of a gene network after hypoxia stress in marine fish. The results provide new clues for further research on the molecular mechanisms underlying hypoxia tolerance in *L. crocea*.

## 1. Introduction

Oxygen is a key environmental factor for growth, development, and reproduction of various living organisms; however, as it is not easily soluble in water, the dissolved oxygen (DO) content of natural water is exceptionally low (~1/34th of that in air). Furthermore, the DO levels in water bodies are susceptible to various natural and human-induced factors, resulting in aquatic animals often suffering from hypoxia effects on their growth, development, and reproduction [1,2]. Today, hypoxia has become one of the most serious and widespread problems in freshwater and marine ecosystems worldwide. Studies show that more than 400 hypoxia zones have emerged in the world’s oceans, covering thousands of square kilometers, with some offshore areas even becoming permanently hypoxic. Moreover, environmental factors such as climate change are set to exacerbate the problem [3,4,5,6]. Hypoxia has altogether caused massive mortality of invertebrates and fish in global waters, altered migration and reproductive cycles, and reduced habitat areas, which seriously affect the stability of the earth’s ecosystem [7]. Imperatively, the effects of hypoxia on aquatic animals have become a hot topic in academic research worldwide.

The large yellow croaker (*Larimichthys crocea*), a marine fish endemic to the southeast coast of China, is named after the croak it makes when spawning. Sought after for its delectable taste and high nutritional value, artificial breeding of *L. crocea* was achieved in 1985, therefore increasing its breeding area and production to become the largest cultured marine fish in China [8]. The limited tolerance of *L. crocea* to hypoxia, especially in net cage culture environments, usually results in significant mortality and huge economic losses [9,10,11]. It is thus vital to investigate the molecular mechanisms underlying hypoxia stress in *L. crocea* to both elucidate its adaptative mechanisms to DO changes and inform the development of selective breeding for hypoxia resistance.

Quantitative real-time PCR (qRT-PCR) and microarray techniques have been widely used for gene expression assessment in aquatic genomics studies [12,13,14]. However, compared to these techniques, the high-throughput RNA sequencing approach (RNA-seq) provides more accurate quantification of gene expression and a view of the entire transcriptome [15,16]. RNA-seq data are now widely used in gene expression studies in aquatic animals and have also been successfully applied in gene co-expression network analysis [17,18,19]. Weighted gene co-expression network analysis (WGCNA) is a method frequently used to explore the complex relationships between genes and phenotypes. In WGCNA, genes with similar expression patterns are likely to be functionally related or members of the same pathway, with specific physiological significance for which hub regulatory genes can be identified [20]. This method has been successfully used to model acute infection of *Pseudomonas plecoglossicida* with *L. crocea* [21]; however, RNA-seq data and co-expression network analysis have not been applied in studies related to hypoxia in *L. crocea*.

In this study, transcriptome samples from different time points and tissues of *L. crocea* under hypoxic stress were used for constructing a co-expressed gene network using WGCNA analysis. In addition, each module gene with the samples was correlated with uncover the hub genes related to hypoxia regulation, with the aim of providing new clues for further research on the molecular genetic mechanisms of hypoxia tolerance in *L. crocea*.

## 2. Results

### 2.1. Illumina Sequencing and Reads Mapping

To explore the changes in *L. crocea* under hypoxia and reoxygenation conditions, the transcriptomes of blood, gills, and liver of *L. crocea* under different hypoxia and reoxygenation stress times (0, 6, 24, 48, 96, and 120 h) were sequenced. A total of 956,902,216 clean reads were obtained from 18 transcriptome samples by sequencing with the Illumina 2500 platform (Supplementary Table S1). The number of clean bases was approximately 142.97 Gb and the amount of mean clean data of each sample was 7.94 Gb. The GC content of the 18 samples was between 43.78% and 50.73%, and the base GC content basically conformed to the normal distribution. More than 95.92% of the bases had a base accuracy of 99%, and more than 91% of bases had a base accuracy of 99.9%. The clean reads of each sample were sequenced and compared with the reference genome of *L. crocea*. The mapping rates from the 18 transcriptome libraries were high, ranging from 76.35% to 86.76%. These results indicate the quality of the transcriptome sequencing data and validate its use in the subsequent data analysis.

### 2.2. Correlation Analysis between Samples

**Figure 1:**
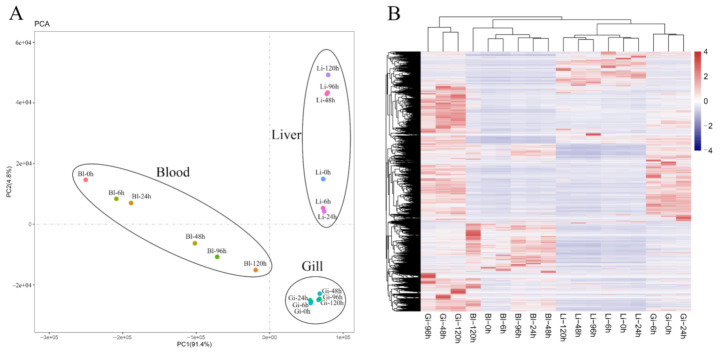
Relationship analysis between samples of different tissues under hypoxia and reoxygenation conditions in *L. crocea*. (**A**) Principal component analysis (PCA) of different transcriptome samples; (**B**) Heat maps analysis of all expressed genes in different samples, values in toolbar are log10 FPKM values of expressed genes.

Principal component analysis (PCA) results of transcriptome samples indicated that the tissue type is the main factor affecting transcriptome results. Sequenced samples were first divided into three sub-categories based on the three tissue types, and then divided into two categories (0 h–6 h–24 h and 48 h–96 h–120 h) based on the time points (Figure 1A). The main expression of the transcriptomes of the 18 samples is shown in Figure 1B. The heat map shows clustering results similar to those of the PCA analysis.

### 2.3. Differentially Expressed Genes (DEGs)

**Figure 2:**
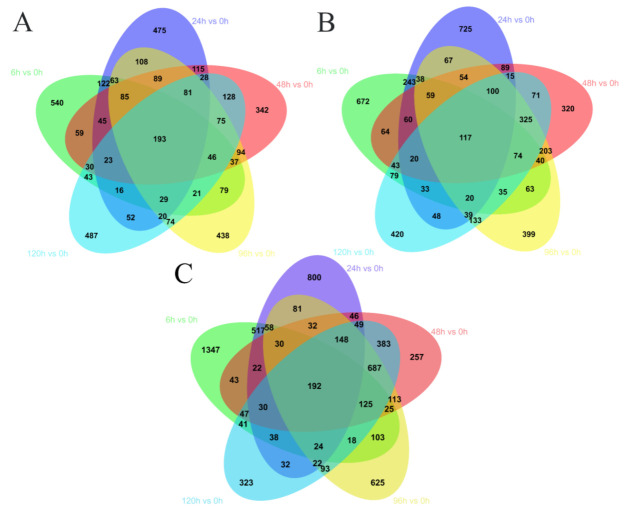
Venn diagrams of differentially expressed genes in the blood (**A**), liver (**B**), and gills (**C**) of *L. crocea* under hypoxia and reoxygenation conditions. All samples were compared to the 0 h control for the respective tissue types.

To analyze the dynamic changes in *L. crocea* during hypoxia and reoxygenation, multiple transcriptome comparisons and subsequent in-depth DEG analyses were performed. In this experiment, 0 h samples of each tissue (liver: Li-0 h; gills: Gi-0 h; and blood: Bl-0 h) were used as the control group; and compared to samples of other time points to screen for DEGs. A total of 14,881 DEGs were screened from 15 analysis groups (Figure 2). There were 4037, 6203, and 4641 DEGs in the blood, gills, and liver, respectively (Supplementary Table S2). The specific numbers of DEGs in each group are shown in Figure 2.

To carry out an in-depth analysis of DEGs, Gene Ontology (GO) and Kyoto Encyclopedia of Genes and Genomes (KEGG) enrichment analysis of the DEGs were performed. In blood, the majority of the five GO terms with the most significant enrichment are related to the function of the nucleosome, as well as the combination and transport of oxygen. The five most significant GO terms enriched in the gills were related to the regulation of osmotic pressure or ion transport, in addition to the combination and transport of oxygen. However, in the liver, after analyzing the first five GO terms that were significantly enriched at various times, it was found that most of the DEGs were enriched in processes related to energy metabolism, as well as immune and antioxidant systems (Supplementary Figure S1).

The KEGG enrichment results showed that a total of nine significantly up or down-regulated pathways were enriched in blood; among which the ribosome pathway was significantly up-regulated at each time point; lipid metabolism-related pathways were significantly down-regulated at Bl-6 h and Bl-24 h; and protein metabolism-related pathways were also significantly down-regulated at Bl-48 h and Bl-96 h (Supplementary Figure S2). For the DEGs in the gills of *L. crocea*, 23 pathways with significant changes were enriched, including eight pathways related to signal transduction, nine pathways related to metabolism, and some related to immune factors and cell cycle (Supplementary Figure S3). Among them, the ECM-receptor interaction was significantly up-regulated at all time points except Gi-24 h. Most of the pathways that were enriched in the liver were related to carbohydrate metabolism, lipid metabolism, and protein metabolism (Supplementary Figure S4).

### 2.4. WGCNA

**Figure 3:**
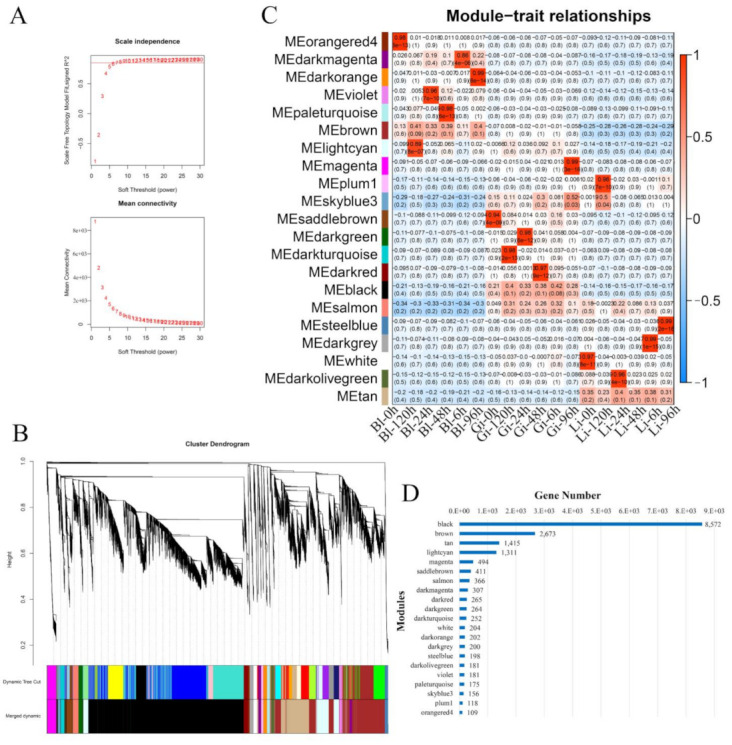
Weighted gene co-expression network analysis (WGCNA) of gene expressions. (**A**) Scale-free topology model fit and gene mean connectivity under different soft threshold power; (**B**) Clustering dendrogram of genes and module division by WGCNA. The 21 co-expression modules are shown in different colors; (**C**) Heat map for the association of gene co-expression network modules with samples; (**D**) Histogram of the number of genes in each module.

WGCNA was performed to identify genes related to hypoxic stress. After filtering out the low-expressed genes (fragments per kilobase of transcript per million; FPKM < 0.01) in 18 transcriptome sequencing libraries, 18,054 effective genes were obtained for WGCNA analysis. The WGCNA packet was used to calculate the weight value to make the network conform to the scale-free network distribution. According to the results in Figure 3A, the soft threshold power β = 8 was selected to construct a co-expression network. The gene co-expression network was then successfully constructed and divided into 21 co-expression modules, with each module using a different color to represent the genes clustered together (Figure 3B). The number of genes varied considerably between modules, with the black module clustering to the highest number of genes (8572) while the orangered4 module contained the fewest (109) (Figure 3D) (Supplementary Table S3).

To identify modules that were significantly associated with hypoxia, module eigengenes was calculated using all genes in each module and then correlated the signature genes with the 18 samples for association analysis (Figure 3C). The closer the correlation between a sample and a module is to the absolute value of 1, the more likely it is that the trait is related to the genetic function of the module. The correlation analysis showed that the orangered4, darkorange, violet and paleturquoise modules were significantly and positively correlated with Bl-0 h, Bl-96 h, Bl-24 h and Bl-48 h, respectively (R^2^ > 0.9, *p <* 0.01). The saddlebrown, darkgreen, darkturquoise, darkred, and magenta modules were significantly and positively correlated with Gi-0 h, Gi-24 h, Gi-120 h, Gi-48 h and Gi-96 h, respectively (R^2^ > 0.9, *p <* 0.01). The plum1, steelblue, darkgrey, white, and olivegreen modules were significantly and positively correlated with Li-120 h, Li-96 h, Li-6 h, Li-0 h, and Li-24 h, respectively (R^2^ > 0.9, *p <* 0.01).

### 2.5. Hub Genes Selections

To elucidate the biological functions of the different modules in hypoxic stress, GO functional enrichment analysis and KEGG pathway enrichment analysis were performed for the 14 modules that were significantly associated with the samples in the previous section.

**Figure 4:**
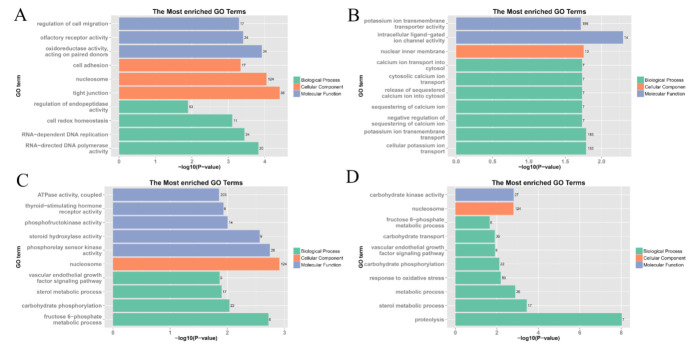
Gene ontology (GO) enrichment analysis of genes within highly correlated modules. (**A**) darkorange module; (**B**) magenta module; (**C**) saddlebrown module; (**D**) darkolivegreen module.

The top 10 enriched terms for each module are shown in Figure 4. The genes in the darkorange module were mainly enriched in four categories: intercellular communication (regulation of cell migration, cell adhesion, tight junction), oxidation-reduction reactions (oxidoreductase activity, cell redox homeostasis), gene replication (nucleosome, RNA-dependent DNA replication, RNA-directed DNA polymerase activity), and other activities (olfactory receptor activity, regulation of endopeptidase activity). Most of the genes in the magenta module are related to ion regulation (potassium ion transmembrane transporter activity, intracellular ligand-gated ion channel activity, calcium ion transport into cytosol, cytosolic calcium ion transport, release of sequestered calcium ion into cytosol, sequestering of calcium ion, negative regulation of sequestering of calcium ion, potassium ion transmembrane transport, and cellular potassium ion transport). Most of the genes in the saddlebrown and darkolivegreen modules are associated with energy metabolism. Some of the other related modules were not significantly enriched (*p* > 0.05) for the KEGG pathway or did not identify effective hub genes, and were therefore excluded for subsequent in-depth analysis.

**Figure 5:**
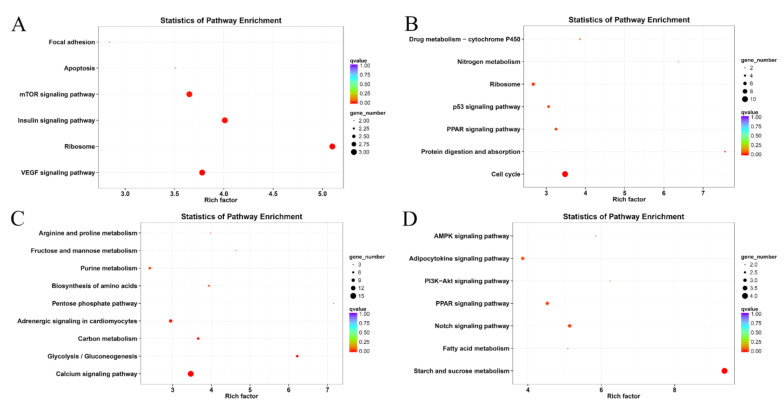
Kyoto Encyclopedia of Genes and Genomes enrichment analysis of genes within highly correlated modules. (**A**) darkorange module; (**B**) magenta module; (**C**) saddlebrown module; (**D**) darkolivegreen module.

The annotation results of the four modules compared to the KEGG database are presented in Figure 5. The enrichment results of pathways showed that the focal adhesion, apoptosis, mammalian target of rapamycin (mTOR) signaling pathway, insulin signaling pathway, ribosome, and VEGF signaling pathways were significantly enriched in the darkorange module. In the magenta module, functions relating to drug metabolism-cytochrome P450, nitrogen metabolism, ribosome, p53 signaling pathway, PPAR signaling pathway, and cell cycle were significantly enriched. In the saddlebrown module, arginine and proline metabolism, fructose and mannose metabolism, purine metabolism, biosynthesis of amino acids, pentose phosphate pathway, adrenergic signaling in cardiomyocytes, carbon metabolism, glycolysis/gluconeogenesis, and calcium signaling pathways were significantly enriched. In the darkolivegreen module, most of the enrichment occurs in metabolism and signaling-related pathways.

**Figure 6:**
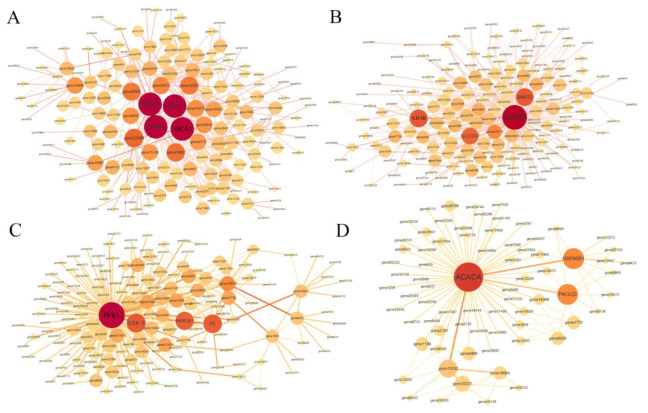
The local co-expression network analysis of darkorange (**A**) magenta (**B**) saddlebrown (**C**), and darkolivegreen (**D**) modules. The size and color of each node circle is positively correlated with the number of interacting genes.

To further determine the function of co-expressed genes within each module and to study hub genes, gene co-expression networks was constructed using Cytoscape (version 3.7.2) software for four selection modules, including darkorange, magenta, saddlebrown, and darkolivegreen modules. The highest degree of co-expressed genes (hub genes) was illustrated with a bigger size and darker color (Figure 6) (Table 1). For example, in the darkorange module, 60S ribosomal protein L8 (*RPL8*), 40S ribosomal protein S16 (*RPS16*), iron-sulfur cluster assembly 1 (*ISCA1*), and erythroid differentiation-related factor 1 (*EDRF1*) were identified as hub genes. In the magenta module, potassium channel subfamily K member 5 (*KCNK5*), sodium-coupled neutral amino acid transporter 2 (*SNAT2*), solute carrier family 22 member 7 (*SLC22A7*) and kinesin-like protein KIF3B (*KIF3B*) were recognized as hub genes. In the saddlebrown module, phosphofructokinase, liver type (*PFKL*), glycogen synthase kinase-3β (*GSK-3β*), pyruvate carboxylase, mitochondrial (*PC*), and alcohol dehydrogenase [NADP(+)] A isoform X1 (*AKR1A1*) were recognized as hub genes. In the darkolivegreen module, acetyl-CoA carboxylase alpha (*ACACA*), heat shock protein 90 kDa beta member 1 (*HSP90B1*), and phosphatidylinositol 4,5-bisphosphate 3-kinase catalytic subunit delta (*PIK3CD*) were identified as hub genes.

### 2.6. Quantitative PCR Validation

**Figure 7:**
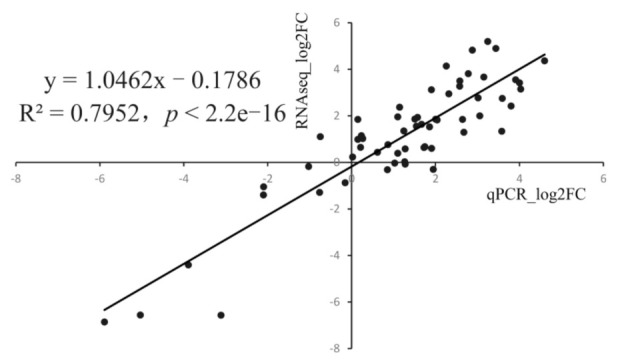
Correlation analysis of Quantitative real-time PCR (qRT-PCR) and RNA sequencing (RNA-seq) results.

To verify the reliability of the transcriptomic data, eight DEGs were selected for qRT-PCR validation, namely citrate synthase (*CS*), glycogen synthase kinase-3α (*GSK3α*), hexokinase-1 (*HK1*), phosphoenolpyruvate carboxykinase (*PEPCK*), 6-phosphofructokinase liver type (*PFKL*), succinate dehydrogenase complex subunits B (*SDHB*), lactate dehydrogenase A (*LDHA*), and triosephosphate isomerase (*TPI*). As shown in Supplementary Figure S5, the qRT-PCR results were generally consistent with the gene expression patterns of the transcriptomic data. Furthermore, correlation analysis also showed a highly significant linear correlation between the results of qRT-PCR and RNA-seq (R^2^ = 0.7952), indicating the high reliability of the transcriptomic data (Figure 7).

## 3. Discussion

The changes in gene expression in *L. crocea* when exposed to acute hypoxia stress are complex, and transcriptome sequencing of these events has not been fully investigated [8,9]. In this study, transcriptome sequencing was used to analyze gene expression in *L. crocea* at different time points and in different tissues (liver, blood, and gills) under hypoxia and reoxygenation stress, and constructed gene co-expression networks together with performing gene network interaction analysis on four of the modules to find the hub genes. The results indicate that hub genes associated with hypoxia and reoxygenation stress may play important roles in identifying candidate biomarkers and understanding the molecular mechanisms of hypoxia tolerance.

### 3.1. Effects of Hypoxia and Reoxygenation on the L. crocea Transcriptome

The PCA and heat map analysis of the three tissues at each time point of hypoxia and reoxygenation stress showed that all three tissues exhibited significant clustering by tissue type rather than time points with significant variation among the samples of each tissue. The results of GO and KEGG enrichment analysis showed that the adaptation strategy of *L. crocea* to hypoxia was significantly tissue-specific, with blood, gills, and liver associated with functional terms and pathways of oxygen transport, ion regulation, and energy metabolism, respectively, which corresponded to the physiological functions of the tissues as well. Similarly, transcriptome analysis of liver, gills, and kidney tissues of *Leuciscus waleckii* under alkaline stress showed different patterns of energy metabolism in different tissues, and DEGs were specific among tissues [22]. The adaptation of gills, liver and muscle tissues of *Cynoglossus semilaevis* to a high-temperature environment was also significantly different, and there was obvious tissue specificity for certain biological functions [23]. The tissue specificity exhibited under hypoxia and reoxygenation stress is mainly due to the presence of tissue-specific genes (luxury genes). However, further investigation is required to explore this in more depth.

Based on the tissue-specific analysis of hypoxia adaptation in *L. crocea*, we expanded on the different functions of the three tissues in hypoxia adaptation. GO analysis and KEGG enrichment analysis were performed on a total of 14,881 DEGs from three tissues, and among these GO terms and pathways, metabolism-related biological process terms were the most enriched, including “carbohydrate metabolism”, “lipid metabolism” and “amino acid metabolism”, followed by oxygen transport-related biological terms in gills and blood, including “oxygen transport”, “hemoglobin complex”, and “transferrin transport”. At the same time, important processes such as “signaling” and “osmolarity regulation” occur frequently. Therefore, this section discusses four aspects of energy metabolism, signal transduction, oxygen transport, and osmotic regulation to provide a comprehensive account of the molecular regulatory mechanisms in *L. crocea* under hypoxia and reoxygenation conditions. As energy metabolism-related topics have been covered in detail in previous articles [9], this discussion will only brush over the most important aspects.

Signal transduction enhances the body’s tolerance to hypoxia by activating transcription or inhibiting translation, which is of great significance for the adaptation of aquatic animals to hypoxia [2,24,25]. This study found that signaling pathways such as the transforming growth factor-β (TGF-β) signaling pathway, insulin signaling pathway, glucagon signaling pathway, ECM-receptor interaction, focal adhesion, and tight junctions were significantly enriched. TGF-β is a signaling pathway mediated by transforming growth factors, which maintains the stability of the cell environment and promotes embryonic development, tissue repair, cell proliferation and differentiation, and immune regulation [26]. It also plays a key role in regulating cell proliferation and is of great significance for maintaining the environmental homeostasis of *L. crocea* tissue under hypoxia [27]. The insulin signaling pathway plays an important role in carbohydrate and lipid metabolism. It can accelerate the transport of glucose into cells, promote the storage of intracellular triglycerides and glucose in the form of glycogen in various tissues, inhibit glycogen decomposition and gluconeogenesis, and maintain the balance of carbohydrate and lipid metabolism [28,29]. Furthermore, glucagon and insulin induce opposite metabolic effects [30]. Therefore, the tissues of *L. crocea* under hypoxia can jointly regulate the expression of genes and proteins related to glycolipid metabolism through the insulin signaling pathway and glucagon signaling pathway, and alter the level of glycolipid metabolism in tissue cells to adapt to the altered metabolic pattern caused by hypoxic stress. Moreover, the ECM is a complex mixture of structural and functional macromolecules that plays an important role in the morphogenesis of tissues and organs as well as maintaining the structure and function of cellular tissues [31,32]. It can interact with a variety of cell surface receptors simultaneously and plays a very important role in intercellular signal transduction. The activation of ECM receptors in a hypoxic environment triggers a series of signals in the cell to cause different gene expression, which may be a key factor for the normal function of *L. crocea* tissue under hypoxic stress [33]. Functionally similar to ECM receptors, focal adhesions and tight junctions are also a type of cell-to-cell connection and involved in the regulation of intercellular signal transduction [34,35]. The above results indicate that under acute hypoxic stress, *L. crocea* can rapidly adapt to the hypoxic environment by modulating these signal transduction pathways and altering the expression of genes related to antioxidants, cell proliferation and differentiation, signal transduction, and glycolipid metabolism in the organism by regulating their target genes.

It is generally hypothesized that the effect of hypoxia on fish ion regulation is minimal. However, some studies have shown that the ion regulation and osmotic pressure balance of fish in a hypoxic environment will be affected to a certain extent. For example, Victoria et al. found that the ion balance of *Gymnocypris przewalskii* was significantly disrupted after 24 h of acute hypoxia stress [36]; similarly, the Na^+^ and Cl^-^ concentrations of *Eptatretus stoutii* decreased significantly under hypoxic conditions [37]. The GO enrichment results in *L. crocea* gills revealed that the five most significantly enriched functional groups at each time point were related to osmotic regulation or ion transport, including ammonium transmembrane transport, zinc ion binding, and organic cation transport, suggesting that hypoxia can affect ion regulation in *L. crocea* gills. A central process in ion regulation is active Na^+^ transport, and ion transport through the sodium-potassium pump on the gill epithelium is therefore energy-intensive as is the energy consumed to excrete chloride ions [38,39]. In this study, it was found that glycolipid metabolism-related pathways such as glycerolipid metabolism, glycolysis/gluconeogenesis, and carbon metabolism were significantly enriched in gills, indicating that the gills of *L. crocea* are greatly affected by osmotic balance under hypoxic stress, and the gills glycolipid metabolism process can provide energy support for ion transport and osmotic regulation. Regarding the effect of hypoxia on the regulation of osmotic pressure in the gills of *L. crocea*, further experimental research is needed on its blood ion concentration as well as specific transport enzymes (such as Na^+^/K^+^-ATPase).

The GO analysis of DEGs in blood revealed that GO terms related to oxygen transport and binding, such as the vascular endothelial growth factor (VEGF) signaling pathway, hemoglobin complex, and transferrin transport, were significantly enriched. In the gills, GO terms such as hemoglobin complexes, oxygen transport viability, and oxygen binding were also significantly enriched. VEGF is a growth factor that can specifically act on vascular endothelial cells and acts as a key signal transducer in physiological and pathological angiogenesis [40]. The binding of VEGF to its specific receptor leads to a series of different signaling pathways, resulting in the up-regulation of genes mediating the proliferation of endothelial cells and vascular permeability [41]. Studies have shown that a significant increase in the expression of VEGF under hypoxic conditions can increase oxygen-carrying capacity [42]. Furthermore, an increase in hemoglobin, a key protein for oxygen binding, in response to hypoxic conditions can also significantly increase the oxygen-carrying capacity of blood [43,44]. Additionally, transferrin (TF) is a non-hemoglobin binding iron β-globin, whose most basic physiological function is to bind and transport Fe^3+^. It binds to the TF receptor to maintain iron homeostasis, and absorb and transport free Fe^3+^ to red blood cells to synthesize hemoglobin, with the remaining Fe^3+^ transported to hepatocytes or macrophages for storage [45,46]. Studies have shown that hypoxia can promote the absorption of iron, i.e., through the up-regulation of serum TF receptor in rats exposed to hypoxia [47]. In addition, Wenger et al. found a significant increase in TF gene expression after incubating HepG2 cells under hypoxic conditions, all of which suggest that up-regulated expression of TF under hypoxic stimulation may play a critical role in hypoxia regulation [48]. Iron is an essential element in many important metabolic processes, and iron-containing proteins are required in processes such as oxygen transport, electron transfer, energy metabolism, and removal of active oxygen. Cells maintain iron homeostasis through iron absorption, storage, and use, and thus maintaining the iron homeostasis in the cell under a hypoxic environment is particularly important for the hypoxia adaptation of *L. crocea* [47,49,50]. As an additional mechanism influencing oxygen metabolism, erythropoietin (EPO), a colony-stimulating factor promotes the differentiation of erythroid-directed stem cells into nucleated erythroid cells capable of synthesizing hemoglobin, and secondarily stimulates the release of reticulocytes and erythrocytes from the bone marrow in vivo [51]. Under hypoxic stimulation, EPO significantly up-regulates gene expression, stimulates the proliferation and differentiation of erythrocytes, and therefore increases the oxygen-carrying capacity of the body to meet the oxygen demand in a hypoxic environment [52].

### 3.2. Functional Analysis of Hub Genes

WGCNA can cluster numerous genes into different co-expression modules according to similarities in gene expression patterns. This allows for selecting expression modules that are highly biologically relevant to the target trait, as well as constructing gene networks to mine the regulated hub genes [53,54]. This has proven to be an efficient data mining method in bioinformatics, and it has been used in *Hexagrammos otaki*, *Ictalurus punctatus*, *Scophthalmus maximus*, *Oplegnathus fasciatus*, *Crassostrea gigas* and other aquatic animals [17,18,19,24,55]. In this study, WGCNA analysis of 18,054 validated genes from 18 transcriptome samples yielded a total of 21 co-expression modules, which were correlated with the samples to select 14 modules that were significantly associated with samples for further study. GO enrichment and KEGG pathway enrichment analyses were then performed on these modules, and four modules, namely darkorange, magenta, saddlebrown and darkolivegreen, were finally obtained.

The darkorange module contained 202 genes, of which 4 hub genes were annotated and 3 of them were DEGs in blood tissues. The hub gene of the darkorange module, *ISCA1*, composed of iron and sulfur ions, not only participate in energy transfer (as the auxiliary group) of electron transfer proteins, but also participate in the biochemical reactions (as the active group) of certain enzymes, redox reactions, DNA replication repair, protein translation, and other regulatory processes [56,57,58]. Thus, *ISCA1* is a key gene in many biochemical processes and of great significance to the hypoxic adaptation of *L. crocea*. *EDRF1* controls the differentiation of both division cycle genes, end undifferentiated genes, and initiate differentiation genes [59,60]. Thus, *EDRF1* may be involved in the proliferation and differentiation of erythrocytes in the blood during hypoxia adaptation in *L. crocea*. Both *RPS16* and *RPL8* are core genes of the darkorange module, and both important components of the mature ribosome structure, which are not only involved in protein synthesis, but also have a variety of extra-ribosomal functions, such as regulating cell growth, proliferation, differentiation, apoptosis, and DNA repair [61,62,63]. This implies that *RPS16* and *RPL8* may not only be involved in hemoglobin synthesis but also play an important role in the proliferation and differentiation of erythrocytes in a hypoxic environment. In addition, results from the KEGG analysis showed significant enrichment of the VEGF signaling pathway, insulin signaling pathway, mTOR signaling pathway, and apoptosis, indicating that oxygen transport under hypoxia adaptation is a complex process that requires the assistance of signal transduction.

The magenta module contained 494 genes, of which 4 were annotated to hub genes. The GO enrichment analysis of the magenta module showed that potassium ion transmembrane transporter activity, intracellular ligand-gated ion channel activity, and other ion regulatory-related terms were significantly enriched. This implies that the magenta module may be related to ion regulation. *KCNK5* which belongs to the G protein-sensitive inwardly rectifying potassium channel family is involved in potassium ion transport in epithelial cells [64]. Correlational studies indicate that *KCNK5* plays an important role in apoptotic volume reduction, cell volume regulation, and regulation of negative membrane potential [65]. This implies that it may be involved in several functions such as apoptosis and ion regulation under hypoxia. Furthermore, both *SNAT2* and *SLC22A7* are transmembrane carriers. *SNAT2* is dependent on the extracellular sodium ion concentration, and its best transport substrate is alanine, the most widely distributed transport protein in the family; *SLC22A7* is a known cotransporter of sodium and phosphate, and can transport organic anions [66,67,68]. Moreover, *KIF3B* is a class of protein macromolecules with cellular motor function and involved in power supply (energy generated by ATP hydrolysis) for intracellular material transport, replication of DNA genetic material, transcription and translation of genetic material, and cell division [69,70]. These functions imply that *KIF3B* may provide transport pathways and power for certain protein complexes in the ion regulation process under hypoxia in *L. crocea*. In addition, KEGG enrichment analysis also showed that pathways such as the p53 and PPAR signaling pathways were significantly enriched, indicating that the magenta module is associated with central signaling which also affects ion regulatory processes under hypoxia.

The saddlebrown module contained 411 genes, of which 4 hub genes were annotated. Energy metabolism is important for hypoxia adaptation in *L. crocea*, and the GO enrichment analysis of genes in the saddlebrown module was significantly enriched for functional modules (ATPase activity, steroid hydroxylase activity, carbohydrate phosphorylation, etc.) mainly related to energy metabolism. In the KEGG enrichment analysis, arginine and proline metabolism, glycolysis/gluconeogenesis, and carbon metabolism were significantly enriched, indicating that energy metabolism plays an important role in the hypoxia adaptation of *L. crocea*. *PFKL* is a rate-limiting enzyme of glycolysis, which is inhibited by high concentrations of ATP and citric acid and activated by adenosine monophosphate and fructose-2,6-diphosphate. Several recent studies have found that hypoxia-inducible factor 1α (*HIF-1α*) in hypoxic environments increases *PFKL* activity by regulating its gene expression to accelerate the rate of glycolysis and thus meet the energy requirements when oxygen supply is insufficient [71,72]. *PFKL* as one of the hub genes, may be important in regulating the rate of glucose metabolism in *L. crocea* in a hypoxic environment. *GSK-3β* is a key enzyme in the glycogen metabolic pathway and plays an important role in the formation of the α-1,4 glucosidic bond [73]. However, *GSK-3β* also plays an important role in the regulation of a variety of cellular functions, including the regulation of cell metabolism and signaling, cell structure and motility, and microtubule motility [74,75]. For example, under hypoxic conditions, suppression of *GSK-3β* expression down-regulates p70S6K1 protein phosphorylation levels and regulates the expression of its downstream signaling molecules HIF-1α and VEGF, therefore inhibiting angiogenesis and apoptosis [76]. *GSK-3β*, a hub gene in the saddlebrown module, may be involved in various aspects of the regulation of hypoxia adaptation in *L. crocea*. *PC*, an important enzyme in glucose metabolism, is expressed in the liver, pancreas, and adipose tissue and is responsible for regulating gluconeogenesis and fatty acid synthesis [77]. In tumor cells, the expression level of *PC* is significantly increased to catalyze the generation of more pyruvate to generate oxaloacetic acid to enter the tricarboxylic acid cycle pathway, thus meeting the energy needs of tumor cells for rapid value addition [78,79]. The growth of tumor cells has been shown to be significantly inhibited when PC expression is inhibited [80]. *PC*, one of the hub genes of the module, may be related to the promotion of glycolysis in a hypoxic environment to accelerate oxidative energy supply. *AKR1A1* catalyzes the conversion of ethanol from ethylene to hydrogen ions and is present in plants, microorganisms, and most mammals. However, it has been shown that *AKR1A1* can convert lactic acid produced by anaerobic metabolism into ethanol in the body and excrete it through the gills under hypoxic conditions in carp such as goldfish, *Carassius auratus,* and *Rhodeus amarus*, thus avoiding acidosis due to excessive accumulation of lactic acid [81,82,83]. As *AKR1A1* was one of the core genes in this module, it implies that *AKR1A1* may have some undiscovered functions or that ethanol metabolic pathways may play a role in *L. crocea* during hypoxia adaptation (requiring the measurement of ethanol levels in their bodies and environment to be certain). These genes have been studied in more detail in mammals and zebrafish, and less so in *L. crocea* necessitating more research.

The darkolivegreen module contained 181 genes, of which 3 hub genes were annotated. *ACACA* is a key enzyme in lipid metabolism that catalyzes the generation of malonyl coenzyme A from acetyl coenzyme A. It is the first rate-limiting enzyme in fatty acid synthesis and plays an important role in carbohydrate and fat metabolism [84]. It has been shown that a significantly high expression of *ACACA* protects cells from apoptosis due to hypoxia and is a key gene in regulating the metabolic state of cells [85]. This implies that *ACACA* may regulate key processes such as lipid metabolism during hypoxia in *L. crocea*. *HSP90B1* is a genetically highly conserved member of the heat shock protein family, and its main function is to act as a molecular chaperone involved in the correct post-translational folding processing of proteins, in addition to anti-apoptosis, regulation of the cell cycle, immune regulation, and participation in cell movement, and migration and other functions [86,87]. Studies have shown that *HSP90B1* is a major regulator of *HIF-1α* activation under hypoxia, suggesting that this gene may be a major regulator of hypoxia stress in *L. crocea* [86]. *PIK3CD*, the main downstream molecule of tyrosine kinases and G protein-coupled receptors, catalyze the production of the second messenger phosphatidylinositol 3,4,5-trisphosphate, and activate numerous downstream activators such as Akt, GSK-3, Forkhead transcription factor FOXO1, and mTOR, thus playing an important role in regulating cell proliferation, differentiation, apoptosis, and glucose transport.

## 4. Materials and Methods

### 4.1. Experimental Materials and Hypoxia Experiments

Large yellow croakers were obtained from the farmed cages in Ningde, Fujian Province, China. The healthy large yellow croakers (weight 63.61 ± 6.63 g, length 15.90 ± 1.52 cm) were randomly selected from the same batch of farmed cages and transported to an indoor concrete pond for 14 days (DO 7.80 ± 0.5 mg/L, 22 ± 0.5 °C). The fish were fed an artificial compound feed containing about 12% moisture, 40% protein, 5% crude fat, 5% crude fiber and 15% crude ash (Fujian Yuehai Feed Co. Ltd., China) twice a day (6:00 a.m. and 5:00 p.m.), and the water was cleaned and changed to maintain water quality. The diet was stopped one day prior to starting experimentation.

The hypoxia experiments were carried out in accordance with the laboratory’s previously described protocols [9]. *L. crocea* were placed in hypoxia water (DO 2.0 ± 0.1 mg/L) for 96 h and then returned to normal DO (7.80 ± 0.1 mg/L) for 24 h. Three *L. crocea* from each of the three parallel tanks were taken at 0, 6, 24, 48, 96, and 120 h. The fish were euthanized with 0.05% MS-222 (3-Aminobenzoic acid ethyl ester methanesulfonate, Sigma, Saint Louis, MO, USA), and their livers and gills were collected on ice trays, snap-frozen with liquid nitrogen, and stored at −80 °C. The blood was drawn from the tail vein, stored in liquid nitrogen, and stored at −80 °C for later use.

The principles and procedures of the sampling methods were in strict accordance with the requirements of the Governing Regulation for the Use of Experimental Animals in Zhejiang Province (Zhejiang Provincial Government Order No. 263, released in 17 August 2009, effective from 1 October 2010) and approved by the Animal Care and Use Committee of Ningbo University.

### 4.2. RNA Sequencing

Total RNA from blood, gills, and liver tissues was extracted at 0, 6, 24, 48, 96, and 120 h using TRIzol (equal mix of RNA from three fish) (Invitrogen, Carlsbad, CA, USA) and recorded as either Bl-0h, Bl-6h, Bl-24 h, Bl-48 h, Bl-96 h, Bl-120 h; Gi-0h, Gi-6h, Gi-24 h, Gi-48 h, Gi-96 h, Gi-120 h; or Li-0h, Li-6h, Li-24 h, Li-48 h, Li-96 h, or Li-120 h, respectively. The concentrations of RNA (OD_260/280_ and OD_260/230_) of each sample were measured using a NanoPhotometer spectrophotometer (Thermo Fisher Scientific, Waltham, MA, USA). RNA integrity and nucleic acid absorption peaks were detected using an Agilent 2100 Bioanalyzer (Agilent Technologies, Santa Clara, CA, USA). RNA samples with sample concentrations ≥ 200 ng/µL, OD_260/280_ between 1.8–2.2, OD_260/230_ ≥ 2.0, and relative intensity noise (RIN) ≥ 7.0, were selected for subsequent library construction.

The above qualified sample (1 µg) was used for cDNA library construction using the NEBNext^®^ Ultra^TM^ RNA Library Prep Kit (Illumina, San Diego, CA, USA). After the libraries passed quality control, the libraries of different samples were pooled according to the effective concentration and the target downstream data volume required, and then the 18 cDNA libraries built were sequenced in both directions using the HiSeq 2500 platform (Illumina, USA), with a sequencing read length of 125 bp at one end and a sequencing volume of 6 Gb per sample.

### 4.3. Data Processing and Analysis

The Fast QC software (version 0.11.8) was used to remove the adapters, unknown bases (ploy-N), and repeat and low-quality fragments from the raw reads obtained by sequencing to obtain high-quality purity data (clean reads) used for subsequent data analysis [88]. The Q20, Q30, and GC content of the clean reads were also counted. The software Hisat2 (version 2.1.0) was used to compare clean reads with the reference genome of *L. crocea* (GenBank assembly accession: GCA_000972845.2) to obtain information about their position on the reference genome and sequence features unique to the sequenced samples [89]. All the raw data have been uploaded to the NCBI website (accession numbers: SRR11300734-SRR11300741 and SRR11300745-SRR11300754).

Before performing differential expression analysis, the clean reads data of each sample was normalized using the edgeR package (version 1.0.0) [90]. Then, the EBseq R package (version 1.30.0) was used to perform differential expression analysis on every two samples, with false discovery rate (FDR) < 0.05, and |log2 (fold change)| ≥ 1 as the threshold for significant differential expression [91]. Genes that met these criteria were identified as DEGs. Then, the GOseq R package (version 1.42.0) and KOBAS were used to perform GO and KEGG significant enrichment analyses for DEGs [92,93].

### 4.4. Weighted Gene Co-Expression Network Analysis

The WGCNA package (version 1.70-3) in R was used to construct a weighted gene co-expression network and divide related modules according to the steps described by Langfelder and Horvath [20]. To ensure that the resulting network is close to the scale-free topology (linear regression model satisfies R^2^ = 0.8), we first use the function in the WGCNA package to calculate the weighting coefficient β. Hierarchical clustering was then performed on 18,054 genes in the 18 transcriptome libraries, and the resulting gene tree map was cut by dynamic cutting to obtain gene collections (modules). The minimum number of genes in a module was set to 100, and modules with similar expression patterns were merged according to the similarity of module eigenvalues (0.75), and other parameters were set according to the default settings.

The eigengene value for each module was calculated using all genes within each module, and correlated this value with each sample. Only *p* values less than 0.05 were considered to be hypoxia-related modules. Genes within the highly related modules were subjected to GO enrichment and KEGG enrichment analyses. Genes with the top 1% kME (module membership) values were used as hub genes, and Cytoscape software (version 3.7.2) was used to construct the visualization network [94].

### 4.5. Quantitative Real-Time PCR Analysis

To verify the accuracy of the RNA-seq results, qRT-PCR was performed on a total of eight DEGs (for some tissue samples with non-DEGs) from three tissues of *L. crocea* using the extracted RNA. The RNA to be tested was first reverse transcribed into cDNA using the PrimeScript^TM^ RT reagent Kit (Takara, Dalian, China) as a template for real-time fluorescent quantitative PCR analysis, followed by quantitative PCR experiments on a Roche Light Cycler 96 using SYBR Green Master I (Roche, Basel, Switzerland). The primers for the target genes were synthesized and provided by BGI Genomics (Shenzhen, China) (see Supplementary Table S4), and the reference gene was *β*-actin. qRT-PCR was performed with three biological replicates per group and three technical replicates, and the results were analyzed using the 2^-ΔΔct^ method.

### 4.6. Statistical Analysis

Statistical analyses were performed using SPSS 19.0 (IBM, Armonk, NY, USA) and Microsoft Excel software. Group means were compared using a one-way analysis of variance. *p <* 0.05 was considered statistically significant.

## 5. Conclusions

**Figure 8:**
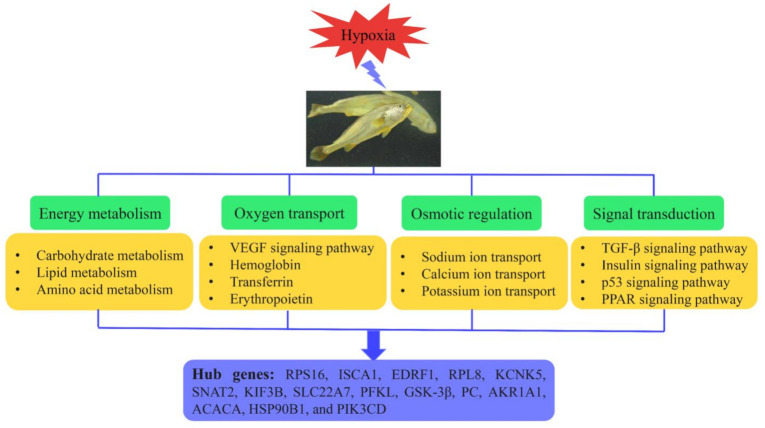
Diagram of hypoxia adaptation strategies and regulatory hub genes in *L. crocea*. See text for abbreviations and explanations.

In summary, *L. crocea* triggers a variety of processes within tissue cells to adapt to hypoxia and reoxygenation environments, including adaptive regulation of energy metabolism, oxygen transport, and ion homeostasis, along with the involvement of various signaling pathways (Figure 8). In addition, some hub genes within the association module were uncovered, such as *RPS16*, *ISCA1*, *EDRF1*, *RPL8*, *KCNK5*, *SNAT2*, *KIF3B*, *SLC22A7, PFKL*, *GSK-3β*, *PC*, *AKR1A1*, *ACACA*, *HSP90B1*, *and PIK3CD*, which could provide clues for further studies on hypoxia adaptation mechanisms in *L. crocea*. In conclusion, this study provides the first phylogenetic insight into the molecular mechanisms underlying the physiological changes in *L. crocea* under hypoxia and reoxygenation stress, which can provide an important reference for subsequent studies on the regulatory mechanisms related to hypoxia tolerance in *L. crocea*.

## Figures and Tables

**Table 1:** Functional annotation of the hub genes of the target module.

Module	Gene Symbol	Gene ID	Description	Degree
darkorange	*RPS16*	EH28_19390	40S ribosomal protein S16	42
darkorange	*ISCA1*	EH28_11232	Iron-sulfur cluster assembly 1	38
darkorange	*EDRF1*	EH28_15699	Erythroid differentiation-related factor 1	37
darkorange	*RPL8*	EH28_17632	60S ribosomal protein L8	37
magenta	*KCNK5*	EH28_04734	Potassium channel subfamily K member 5	118
magenta	*SNAT2*	EH28_19256	Sodium-coupled neutral amino acid transporter 2	58
magenta	*KIF3B*	EH28_23591	Kinesin-like protein KIF3B	56
magenta	*SLC22A7*	EH28_16974	Solute carrier family 22 member 7	48
saddlebrown	*PFKL*	EH28_06053	phosphofructokinase, liver type	97
saddlebrown	*GSK-3β*	EH28_10886	Glycogen synthase kinase-3 beta	42
saddlebrown	*PC*	EH28_15368	Pyruvate carboxylase, mitochondrial	40
saddlebrown	*AKR1A1*	EH28_24096	Alcohol dehydrogenase [NADP(+)] A isoform X1	38
darkolivegreen	*ACACA*	EH28_08273	Acetyl-CoA carboxylase alpha	56
darkolivegreen	*HSP90B1*	EH28_10845	Heat shock protein 90 kDa beta member 1	20
darkolivegreen	*PIK3CD*	EH28_12563	Phosphatidylinositol 4,5-bisphosphate 3-kinase catalytic subunit delta	19

## Data Availability

All the raw data have been uploaded to the NCBI website (accession numbers: PRJNA612352).

## Supplementary Materials

The following are available online at https://www.mdpi.com/article/10.3390/ani11113021/s1. Figure S1: Top GO enrichment results of DEGs at different time points in the blood (A), gills (B), and liver (C) of *L. crocea* (The first 5 terms were selected according to KS values), Figure S2: An overview of the KEGG pathways significantly enriched in differentially expressed genes in the blood, Figure S3: An overview of the KEGG pathways significantly enriched in differentially expressed genes in the gills, Figure S4: An overview of the KEGG pathways significantly enriched in differentially expressed genes in the liver, Figure S5: Validation RNA-seq profiles by qRT-PCR. Relative mRNA expression valves (*n* = 3). Data were expressed as mean±standard deviation, Table S1: Summary of the Illumina sequencing and mapping statistics of the 18 transcriptome samples, Table S2: Differentially expressed genes under different time and tissues of *L. crocea* (FDR < 0.05 and |log2 (fold change)| > 1), Table S3: Detailed list of gene members of each module analyzed by WCCNA in *L. crocea*, Table S4: The sequence of qRT-PCR specific primers of differentially expressed genes in *L. crocea.*

## Abbreviations

ACACA	acetyl-CoA carboxylase alpha
AKR1A1	alcohol dehydrogenase [NADP(+)] A isoform X1
CS	citrate synthase
DEGs	differentially expressed genes
DO	dissolved oxygen
EDRF1	erythroid differentiation-related factor 1
EPO	erythropoietin
FDR	false discovery rate
GO	gene ontology
GSK3α/β	glycogen synthase kinase 3α/β
HIF-1α	hypoxia-inducible factor 1α
HK1	hexokinase-1
HSP90B1	heat shock protein 90 kDa beta member 1
ISCA1	iron-sulfur cluster assembly 1
KCNK5	potassium channel subfamily K member 5
KEGG	Kyoto Encyclopedia of Genes and Genomes
KIF3B	kinesin superfamily protein 3B
LDHA	lactate dehydrogenase A
PC	pyruvate carboxylase
PCA	principal component analysis
PEPCK	phosphoenolpyruvate carboxykinase
PFKL	phosphofructokinase, liver type
PIK3CD	phosphatidylinositol 4,5-bisphosphate 3-kinase catalytic subunit delta
qRT-PCR	quantitative real-time PCR
RIN	relative intensity noise
RNA-seq	RNA sequencing
RPL8	60S ribosomal protein L8
RPS16	40S ribosomal protein S16
SDHB	succinate dehydrogenase complex subunits B
SLC22A7	solute carrier family 22 member 7
SNAT2	sodium-coupled neutral amino acid transporter 2
TF	transferrin
TGF-β	transforming growth factor-β
TPI	triosephosphate isomerase
VEGF	vascular endothelial growth factor
WGCNA	weighted gene co-expression network analysis
