# Supplementary material for: Genetics Responses to Hypoxia and Reoxygenation Stress in Larimichthys crocea Revealed via Transcriptome Analysis and Weighted Gene Co-Expression Network
Source: Animals (Basel). 2021 Oct 20;11(11):3021. doi: 10.3390/ani11113021 (PMC8614329; doi:10.3390/ani11113021)
Supplement: Supplementary file 1 [file animals-11-03021-s001.zip › Files/Supplementary Table S1.pdf]

Table S1. Summary of the Illumina sequencing and mapping statistics of the 18 transcriptome samples.

Sample name	Clean reads	Clean bases (Gb)	GC-Content (%)	Q20 (%)	Q30 (%)	Total mapped (%)	Uniquely mapped (%)
Bl-0h	60,502,530	9.04	48.55	97.42	93.41	85.63	61.41
Bl-6h	50,833,126	7.06	48.96	96.40	91.72	83.86	63.67
Bl-24h	42,253,312	6.32	48.32	96.55	92.01	83.71	68.21
Bl-48h	77,754,614	11.63	48.94	98.24	95.03	85.23	70.30
Bl-96h	58,742,354	8.77	48.11	97.90	94.51	84.41	72.00
Bl-120h	79,925,490	11.94	49.86	97.40	93.52	78.45	68.55
Gi-0h	45,659,176	6.59	44.45	95.93	91.00	80.23	73.30
Gi-6h	47,924,830	7.70	43.78	95.92	91.01	80.20	73.61
Gi-24h	44,323,268	6.69	44.65	96.26	91.63	80.50	73.80
Gi-48h	50,169,538	7.51	50.70	98.16	94.78	86.00	77.93
Gi-96h	41,228,374	6.16	49.21	97.05	93.08	76.35	69.87
Gi-120h	57,811,174	8.65	50.11	97.78	93.95	85.29	78.05
Li-0h	46,205,750	6.90	47.42	96.24	91.47	85.44	78.39
Li-6h	50,003,174	7.45	47.14	96.16	91.34	84.10	77.38
Li-24h	46,838,274	6.97	45.60	96.24	91.53	85.08	78.37
Li-48h	47,288,278	7.08	50.73	98.25	95.15	86.19	78.66
Li-96h	59,428,234	8.89	50.55	98.36	95.43	83.38	76.05
Li-120h	47,411,722	7.09	50.57	98.26	95.16	86.76	79.28
Total	956,902,216	142.97					
