# Supplementary figures and images for: Genetics Responses to Hypoxia and Reoxygenation Stress in Larimichthys crocea Revealed via Transcriptome Analysis and Weighted Gene Co-Expression Network
Source: Animals (Basel). 2021 Oct 20;11(11):3021. doi: 10.3390/ani11113021 (PMC8614329; doi:10.3390/ani11113021)

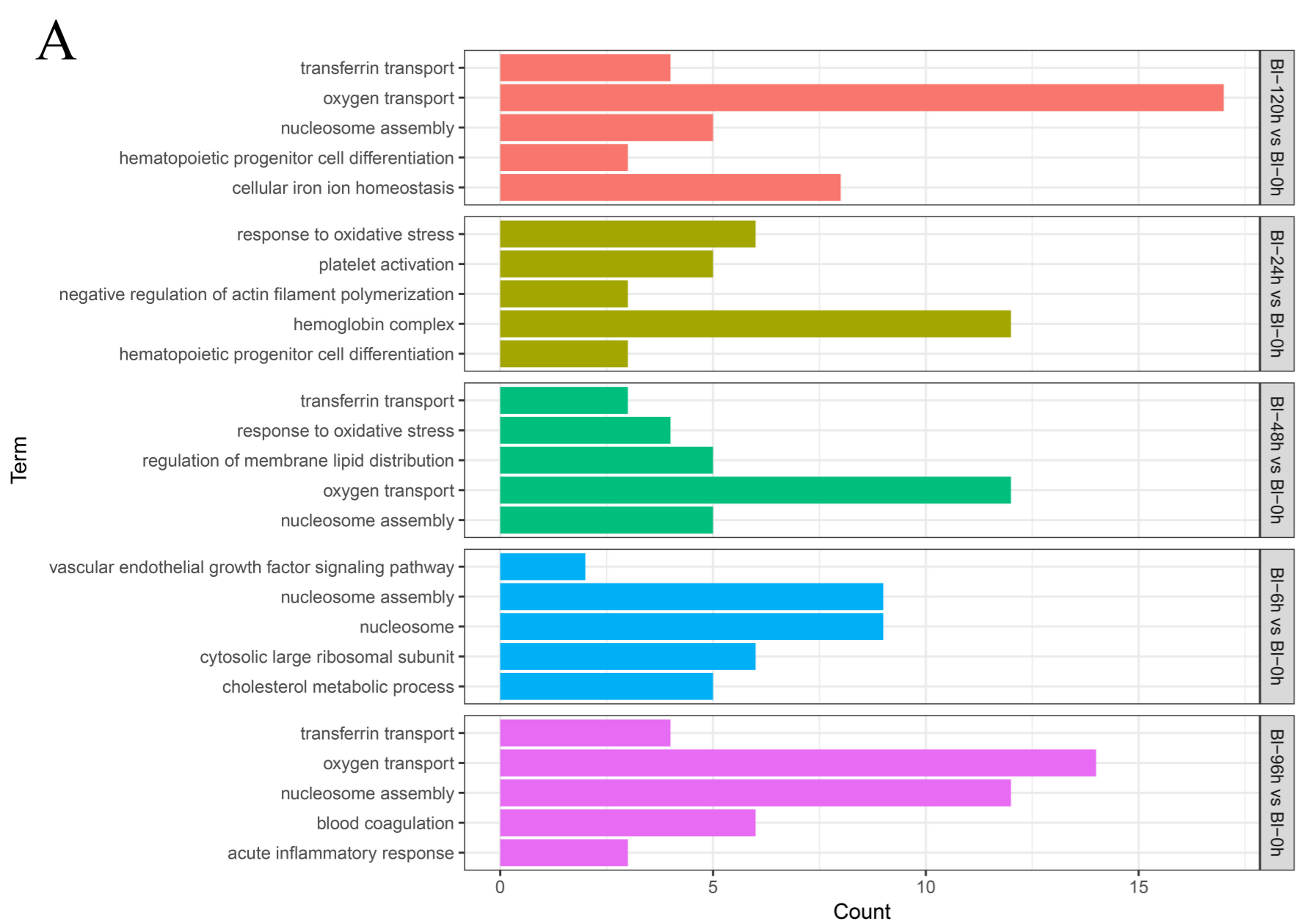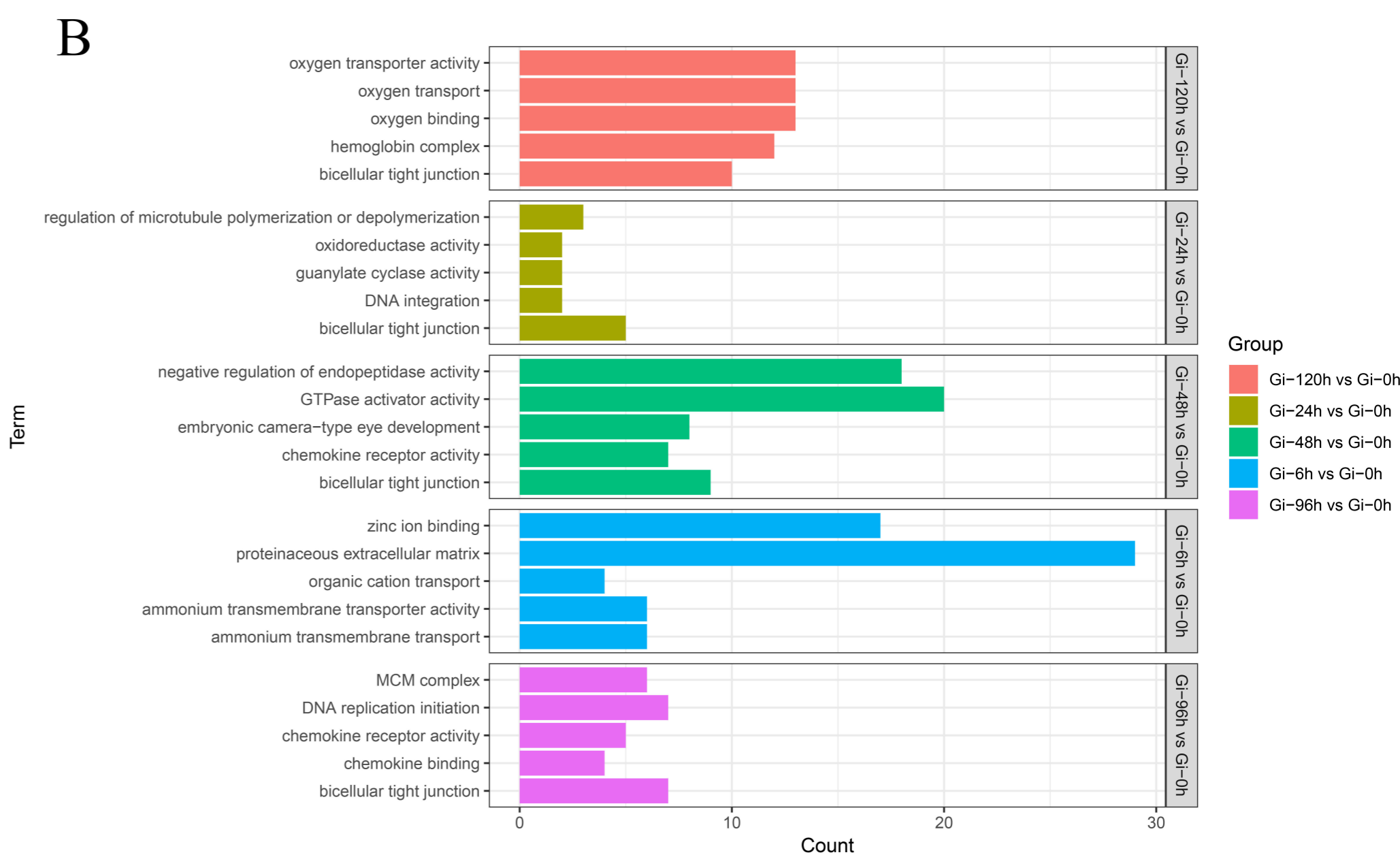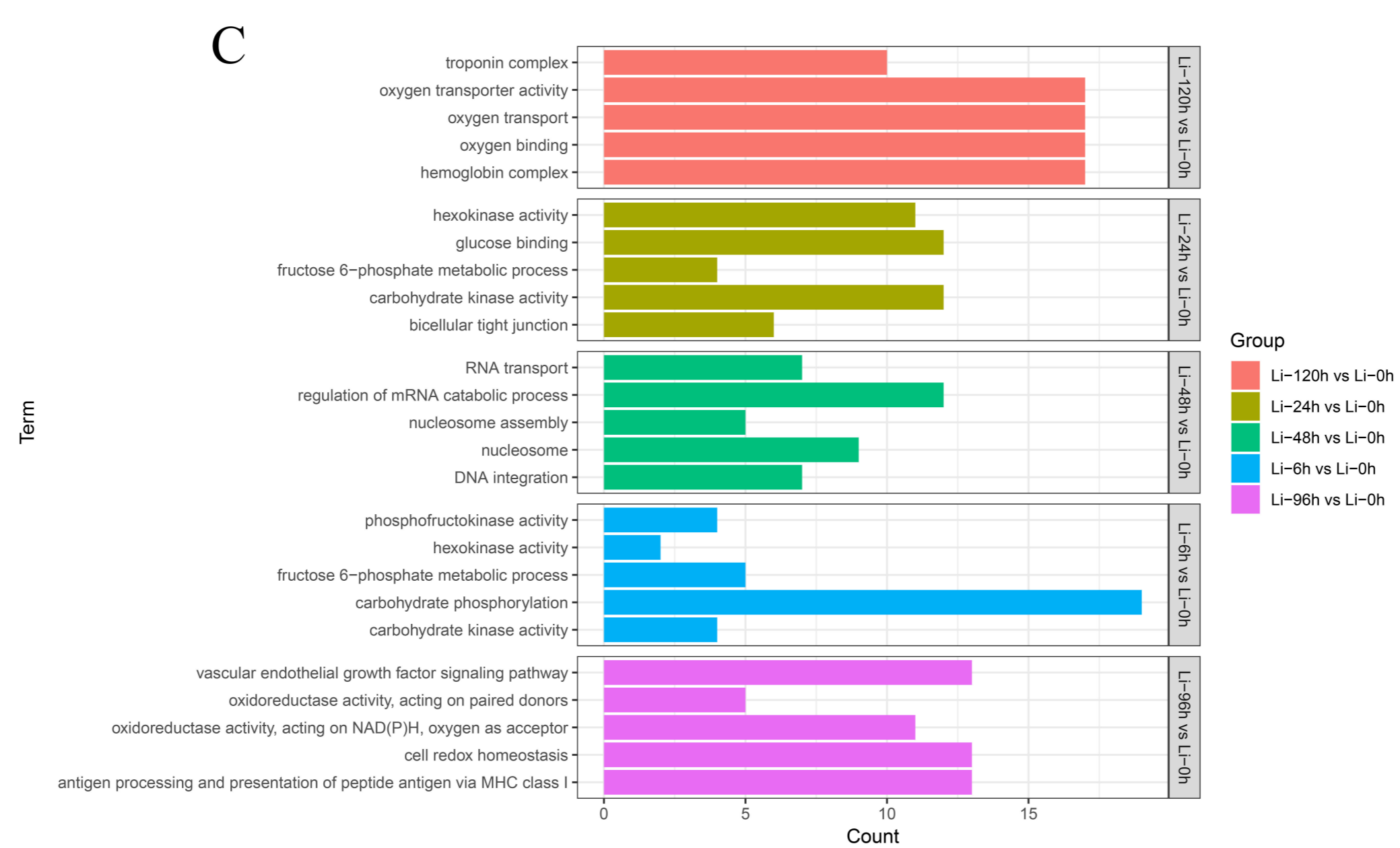

Supplement: Supplementary file 1 [file animals-11-03021-s001.zip › Files/Supplementary Figure S1.pdf]

A

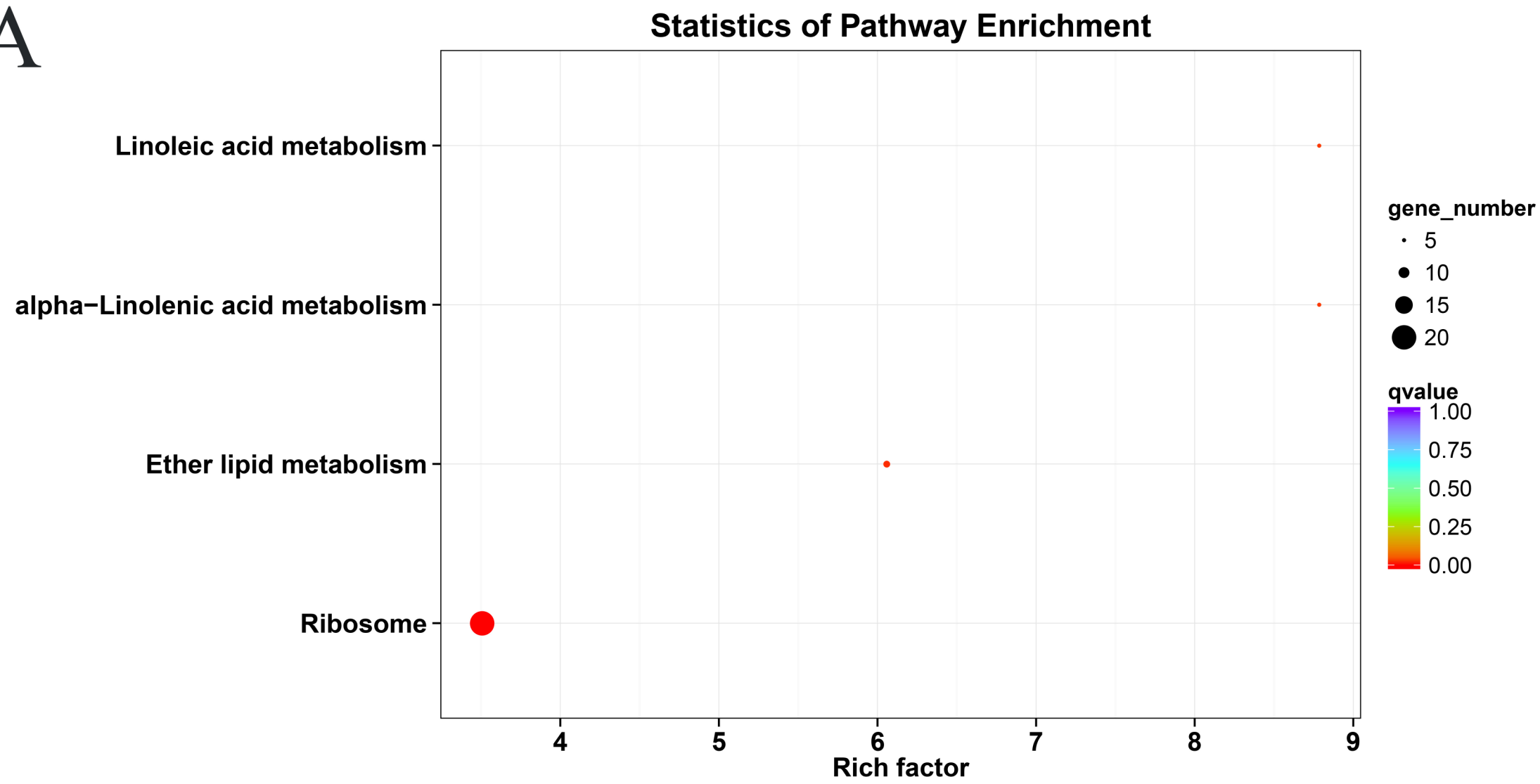

B

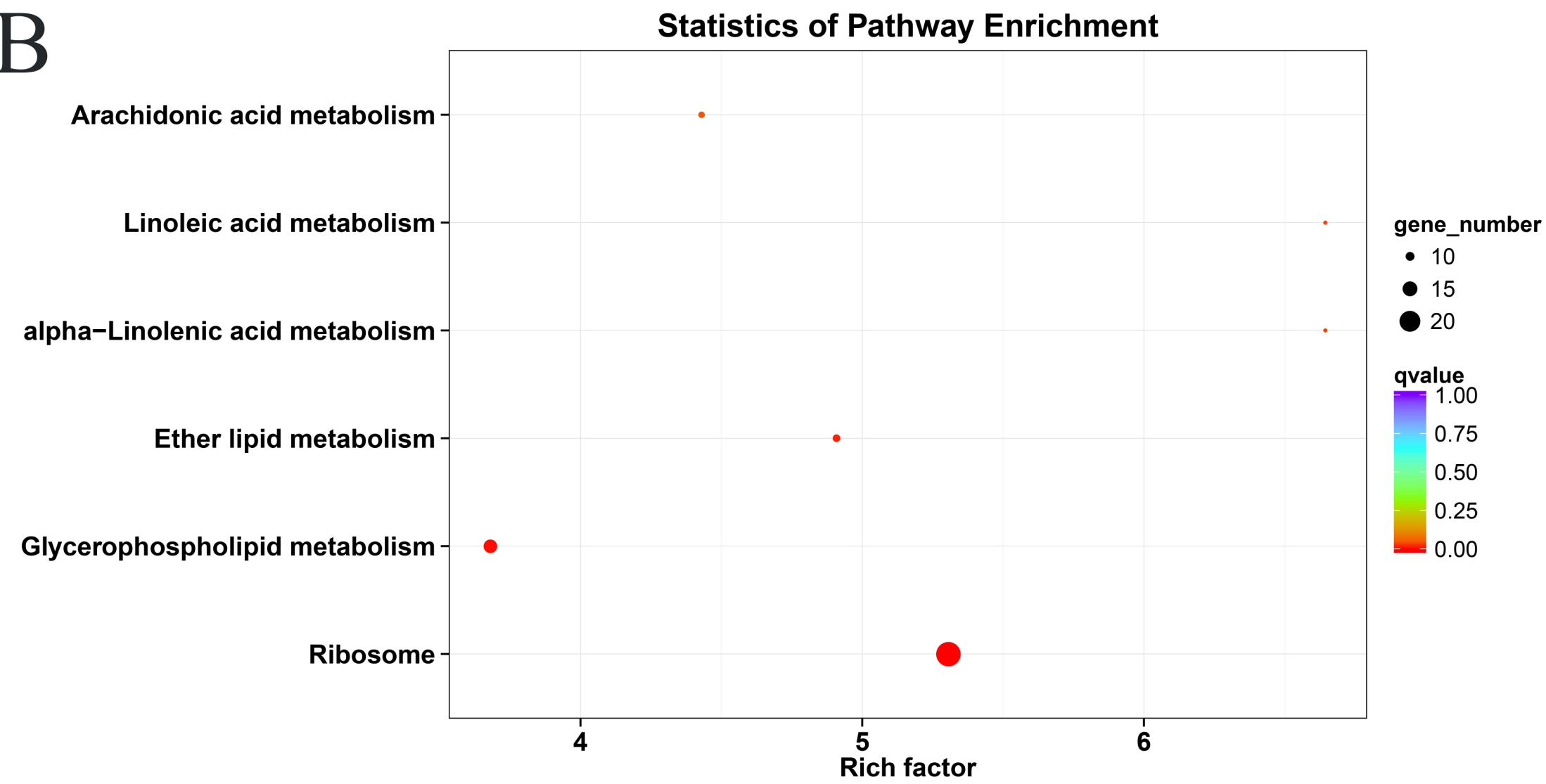

C

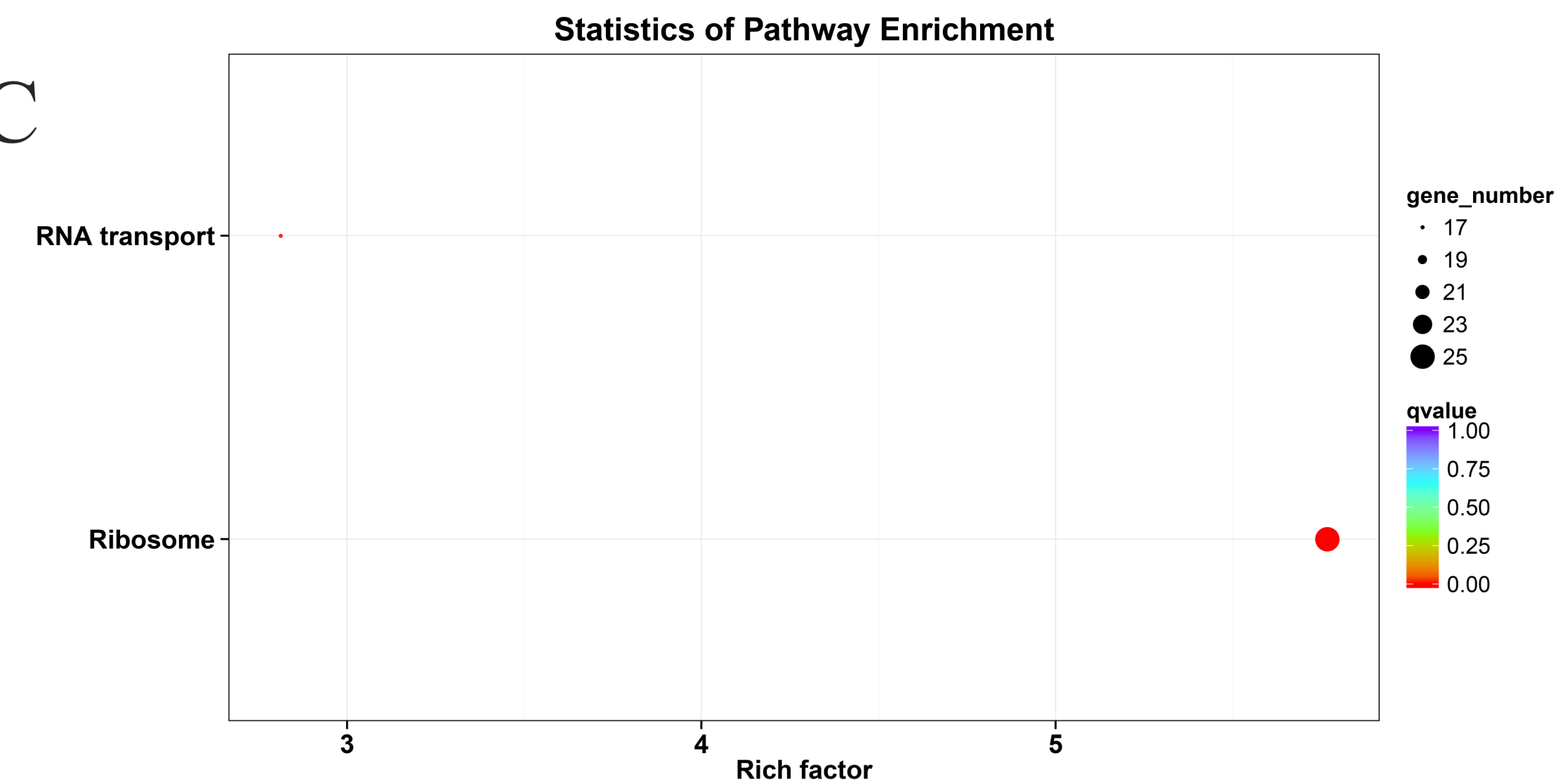

D

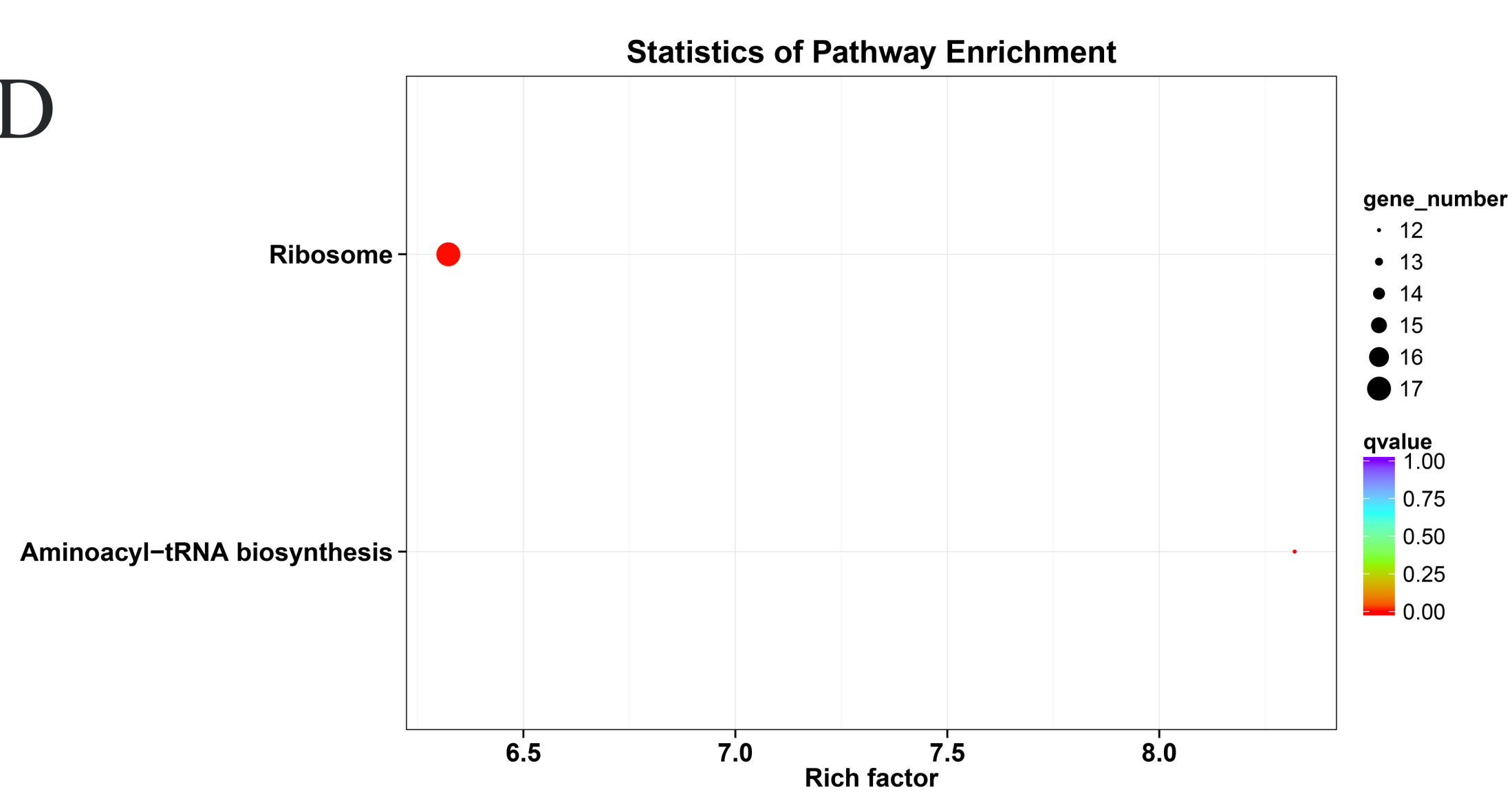

E

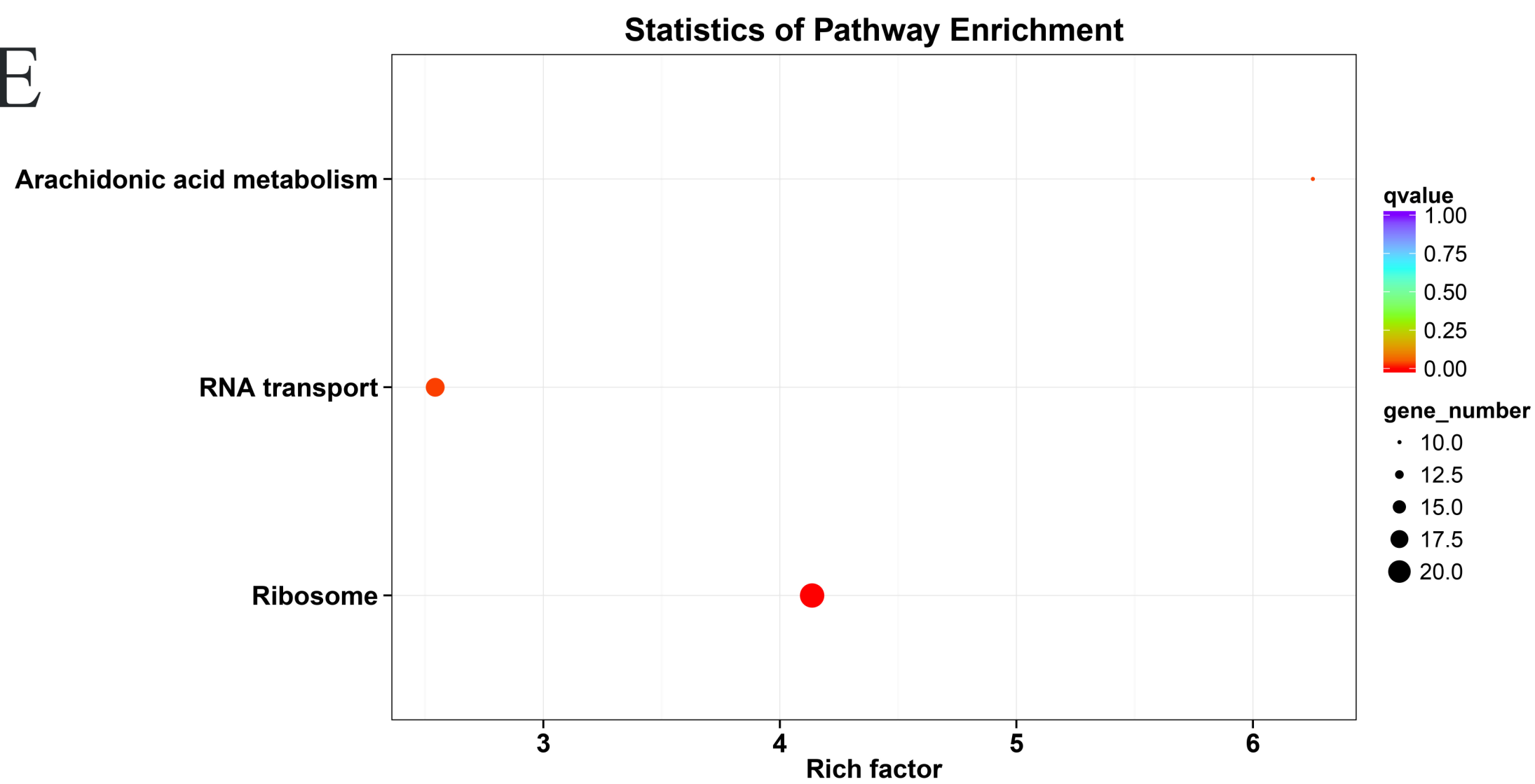

Supplement: Supplementary file 1 [file animals-11-03021-s001.zip › Files/Supplementary Figure S2.pdf]

A

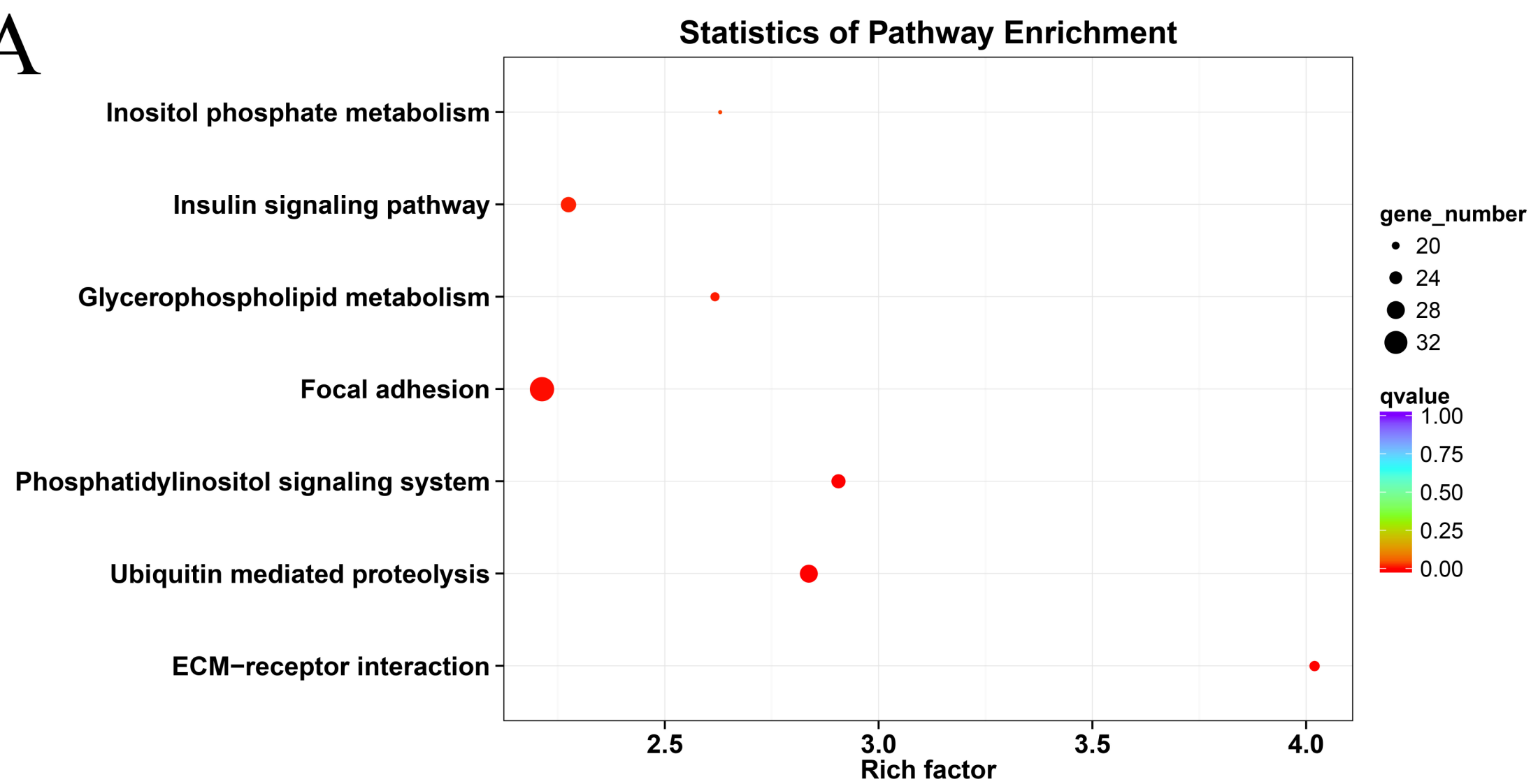

B

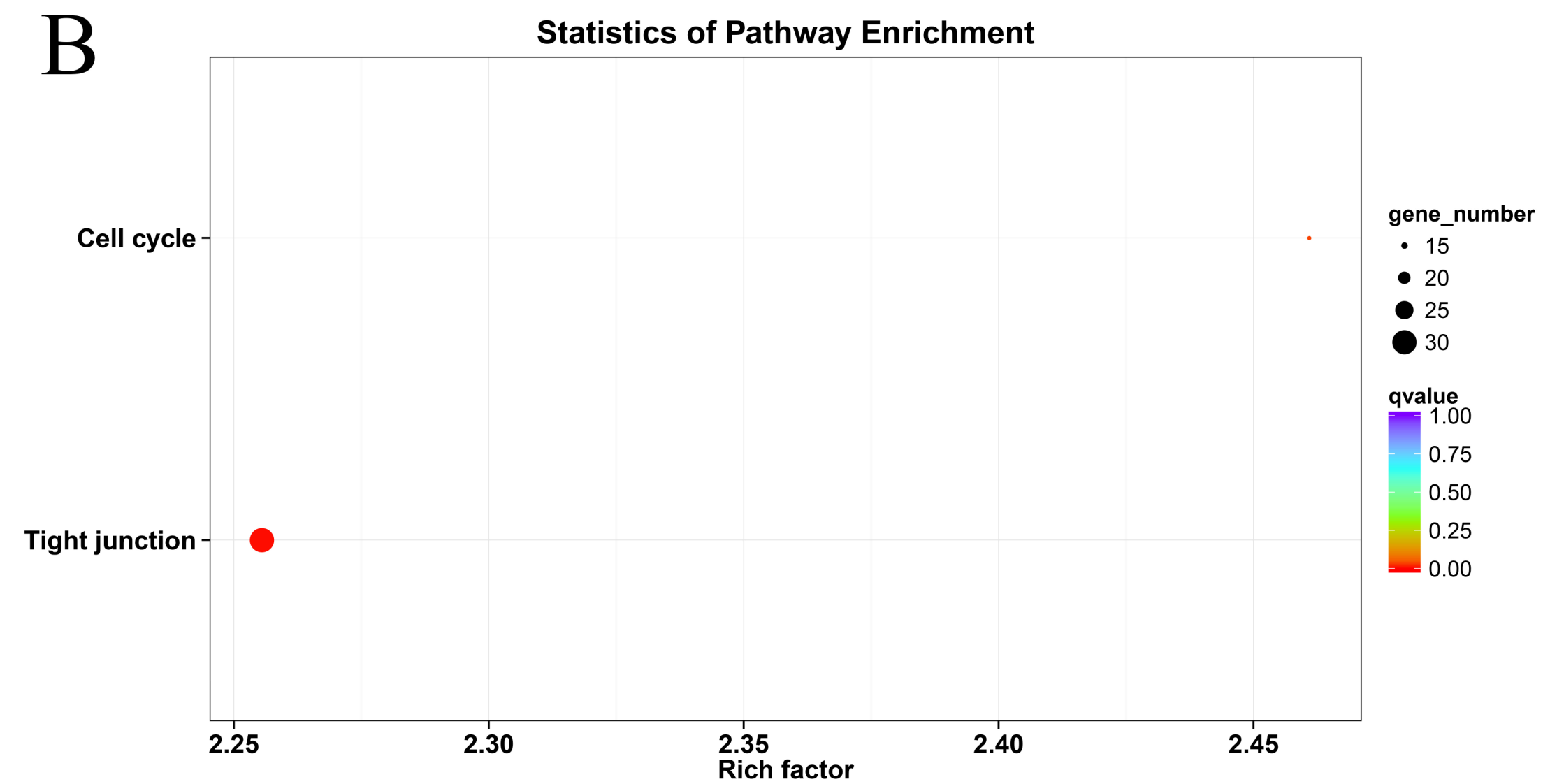

C

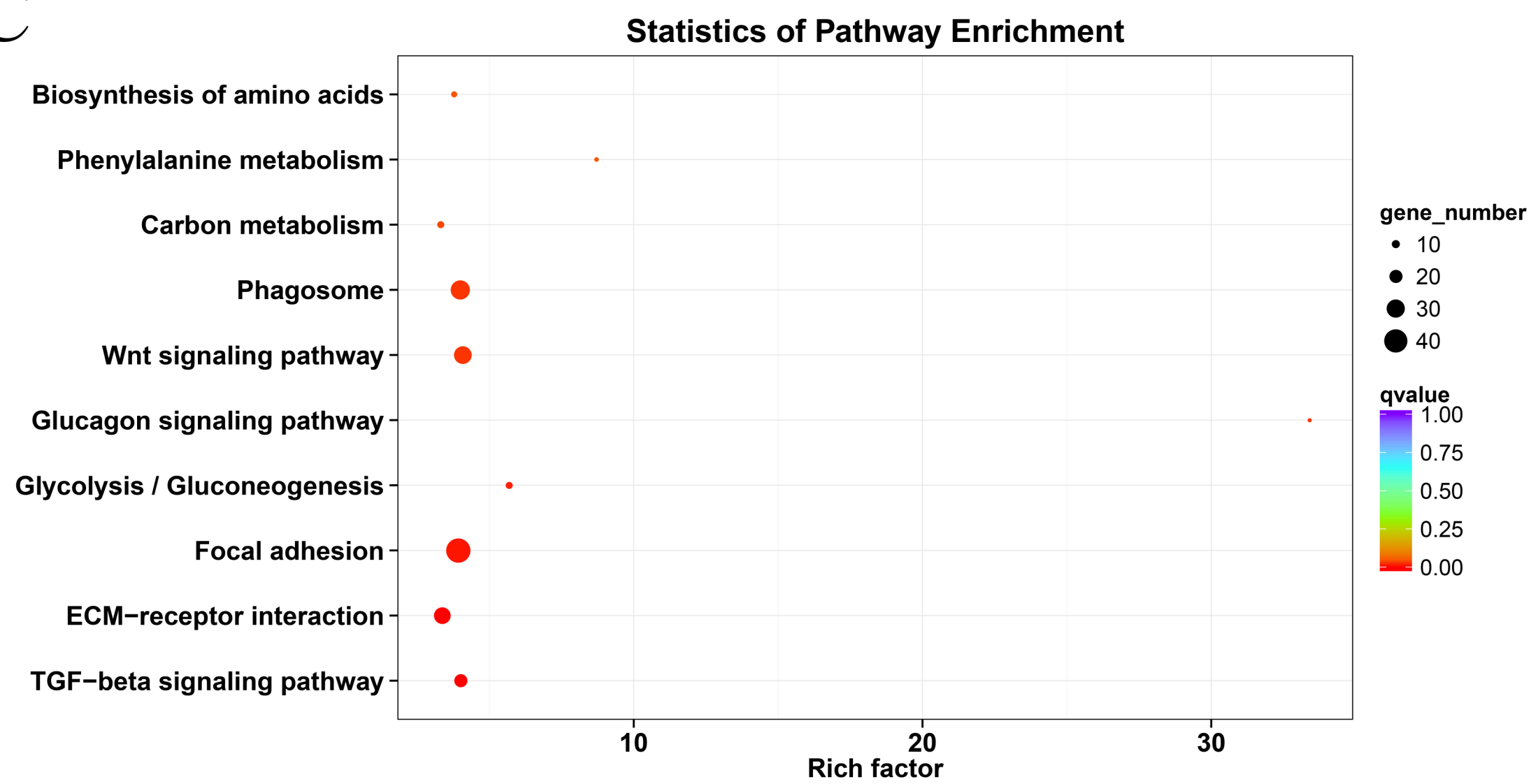

D

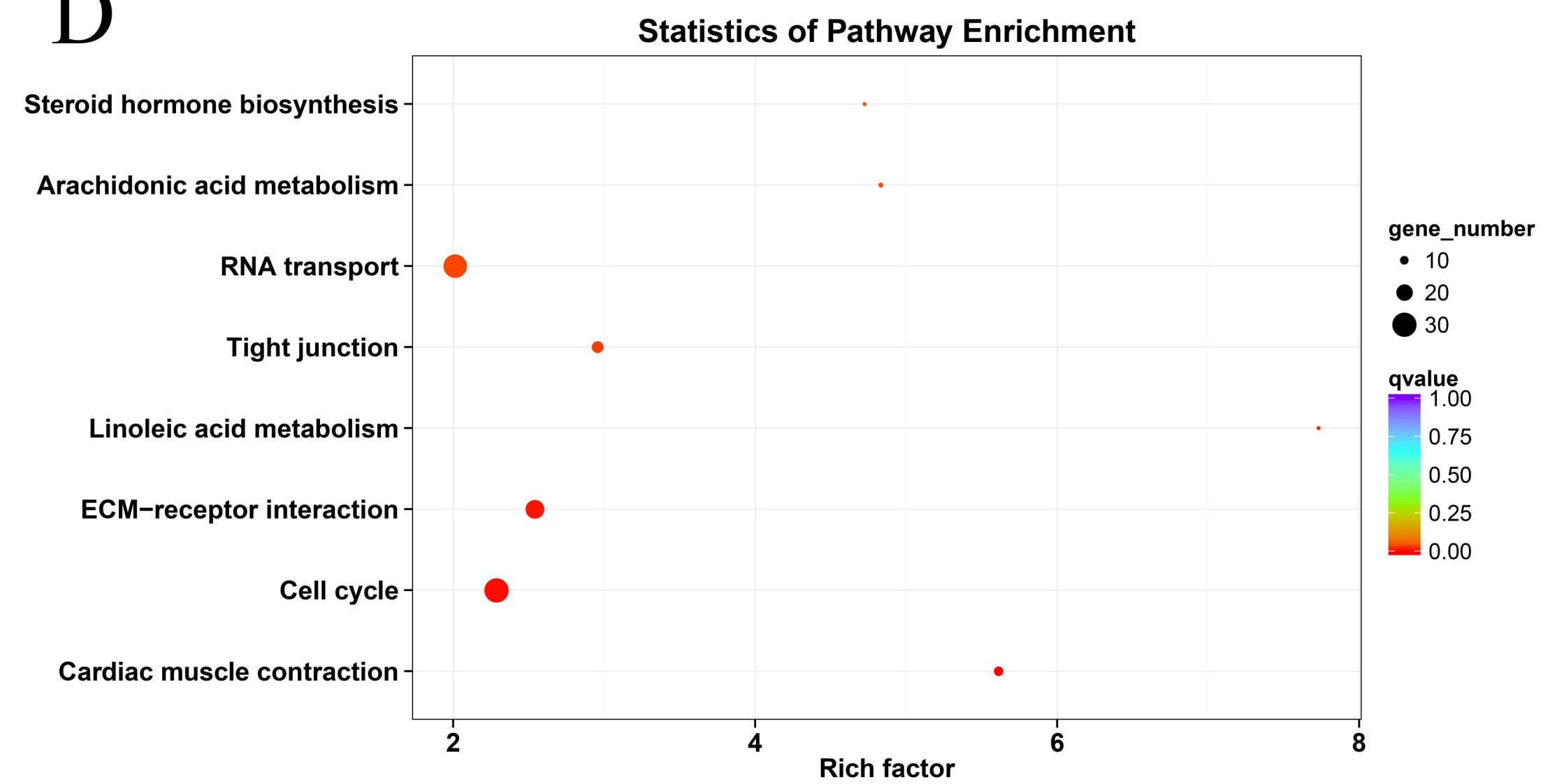

E

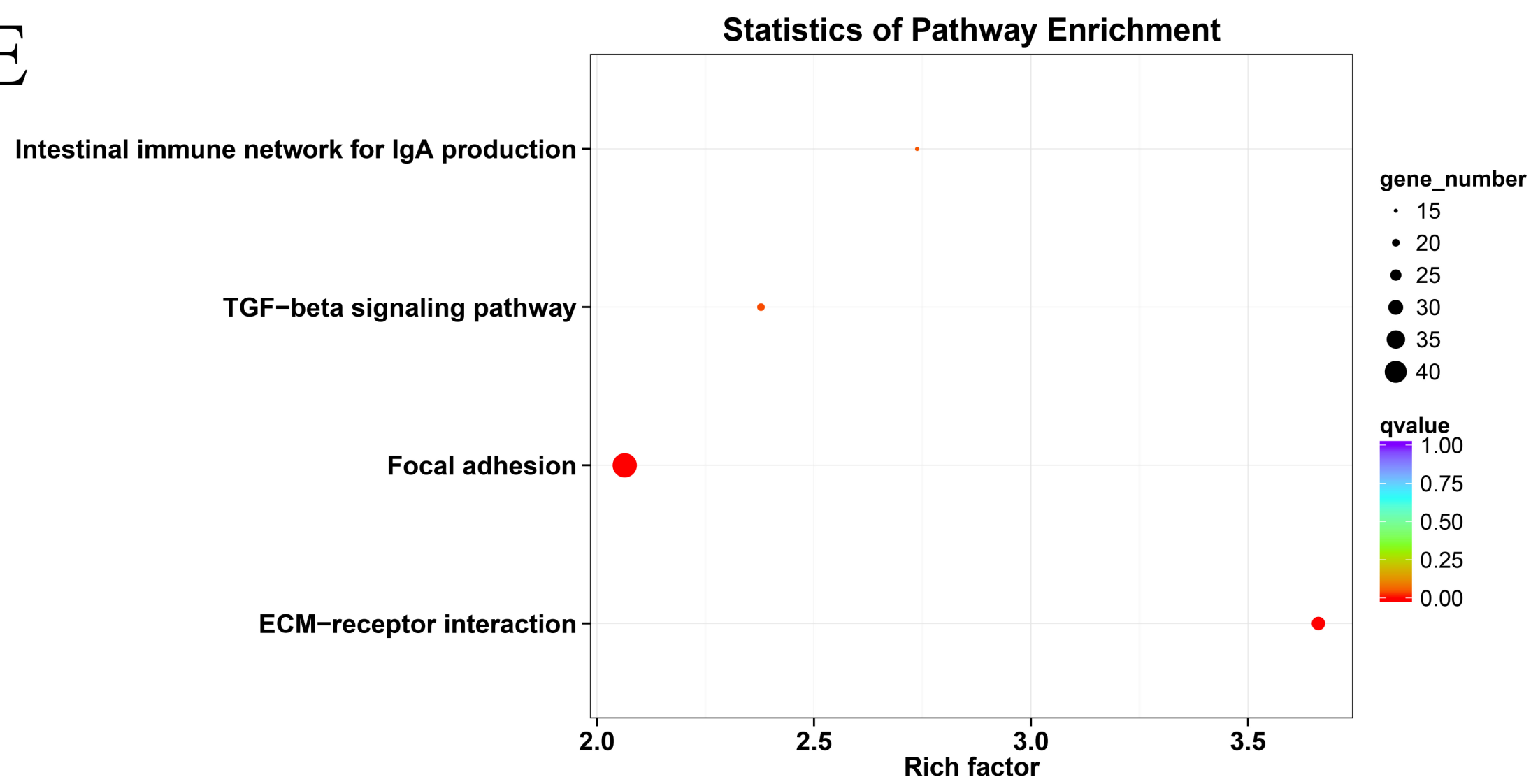

Supplement: Supplementary file 1 [file animals-11-03021-s001.zip › Files/Supplementary Figure S3.pdf]

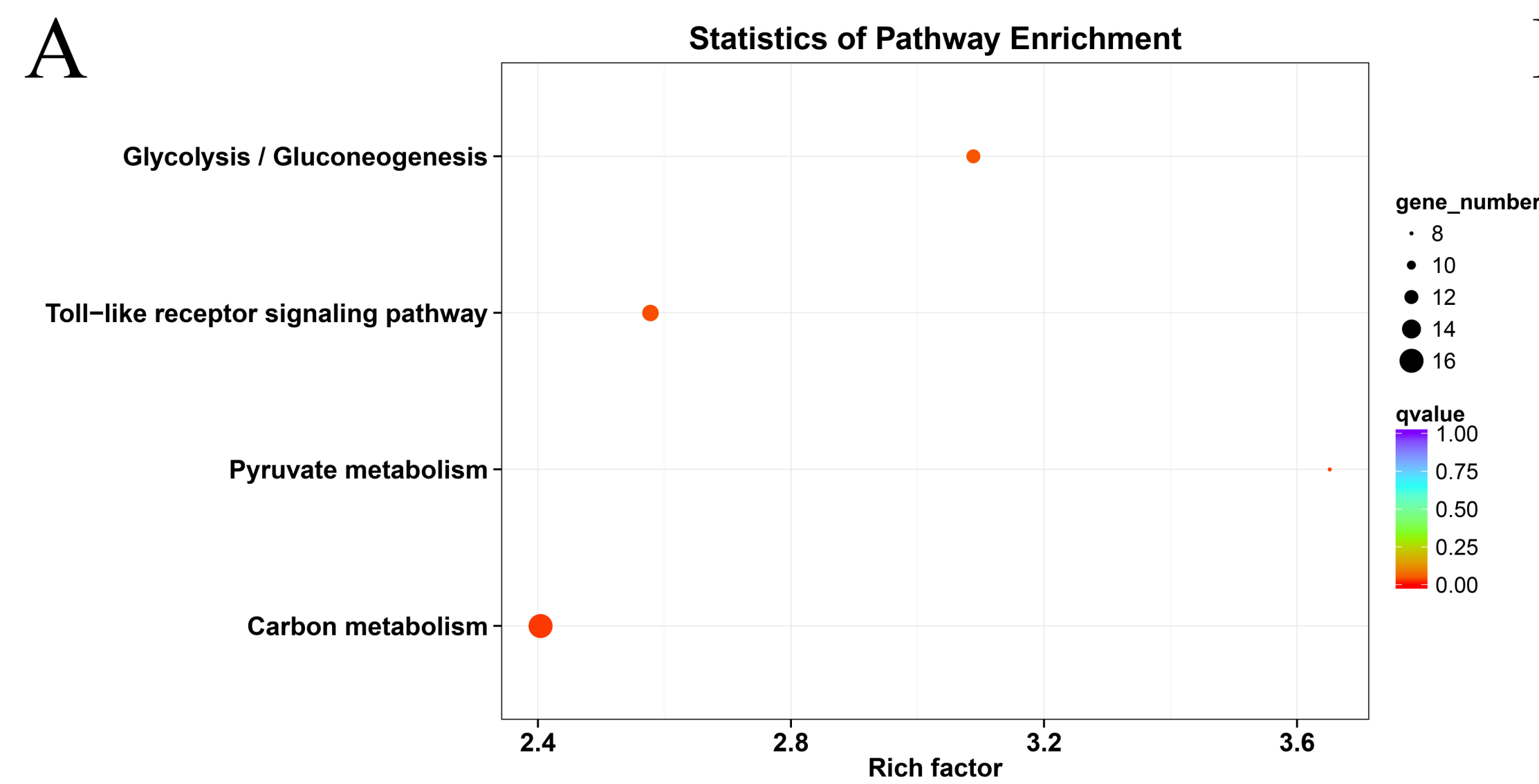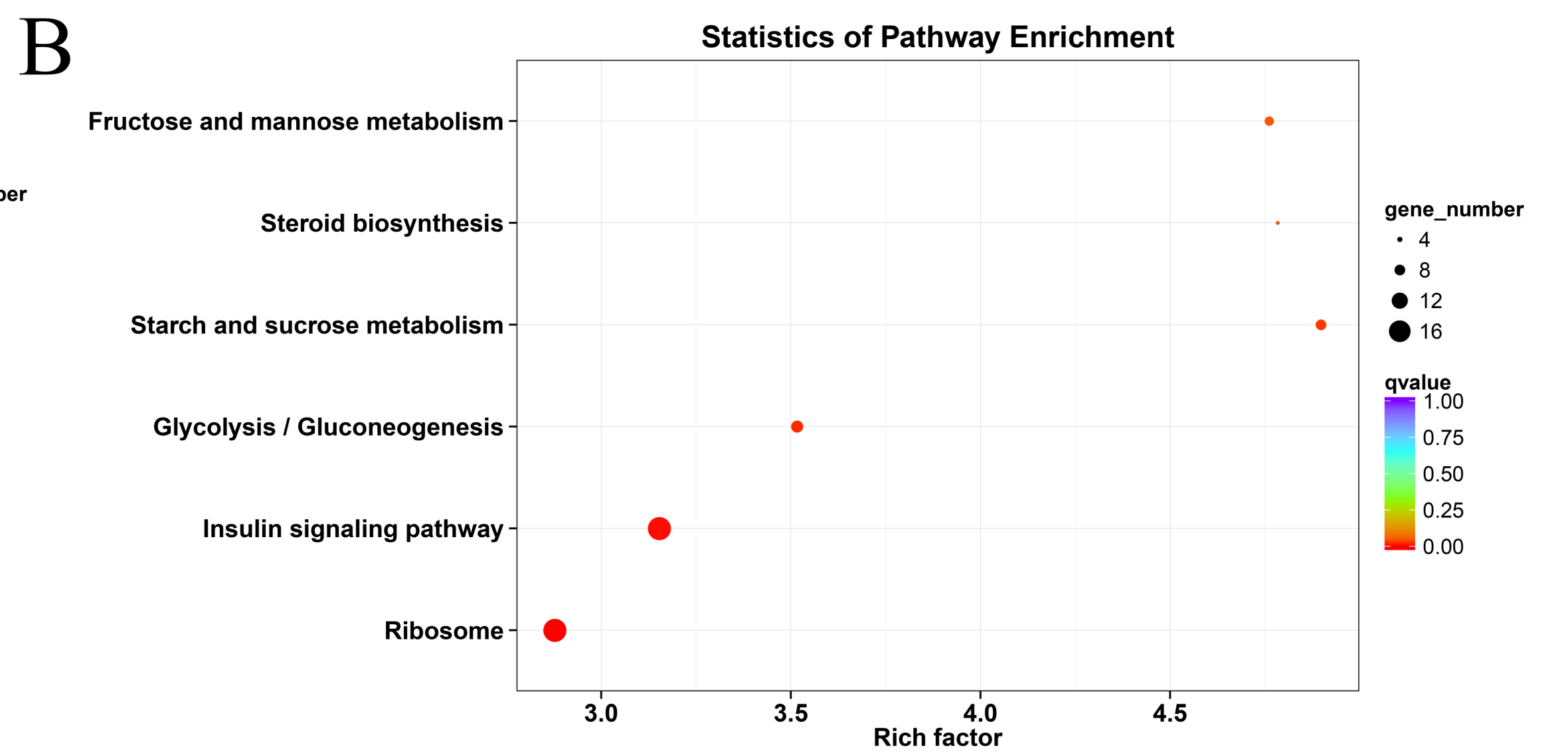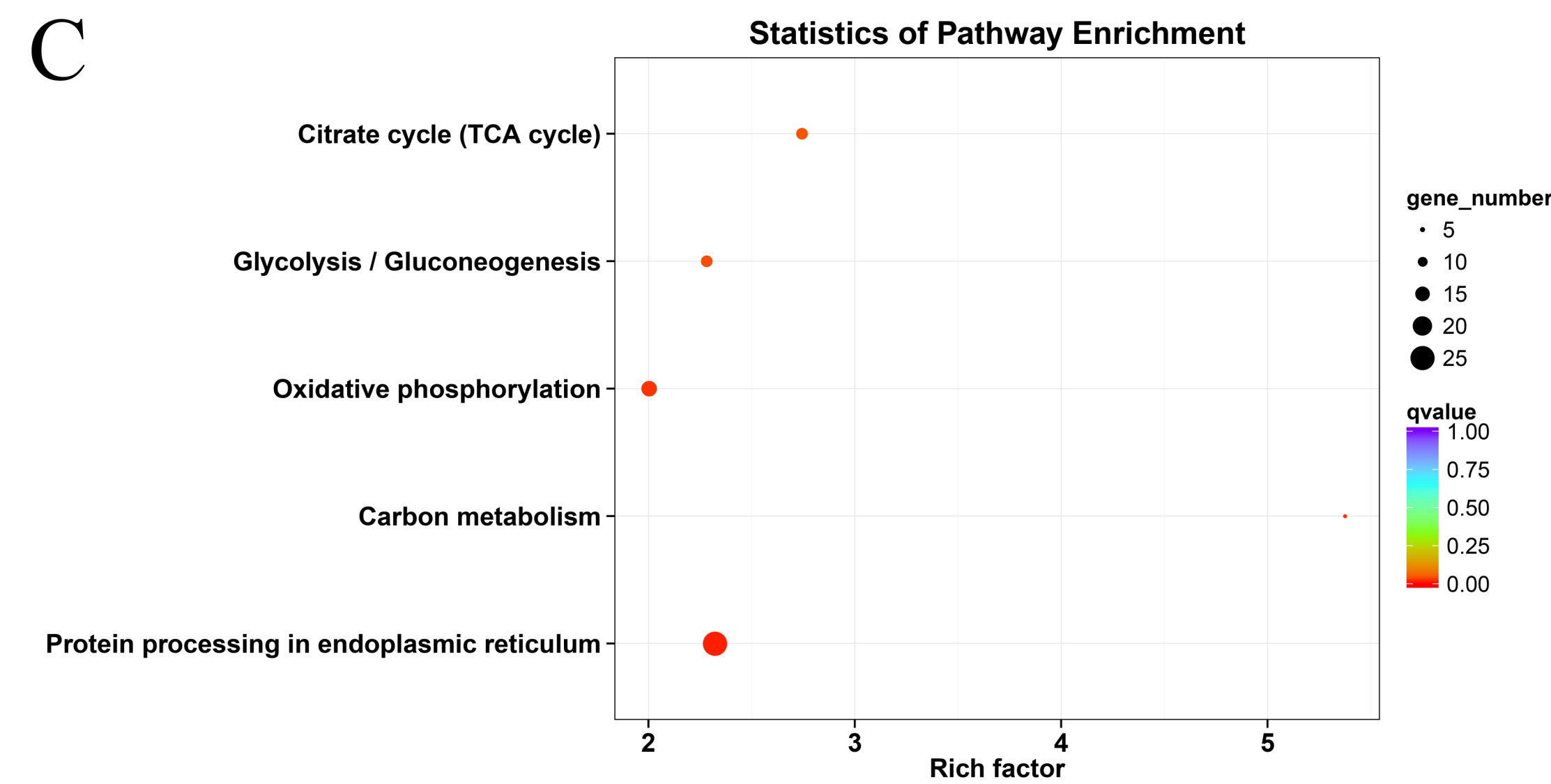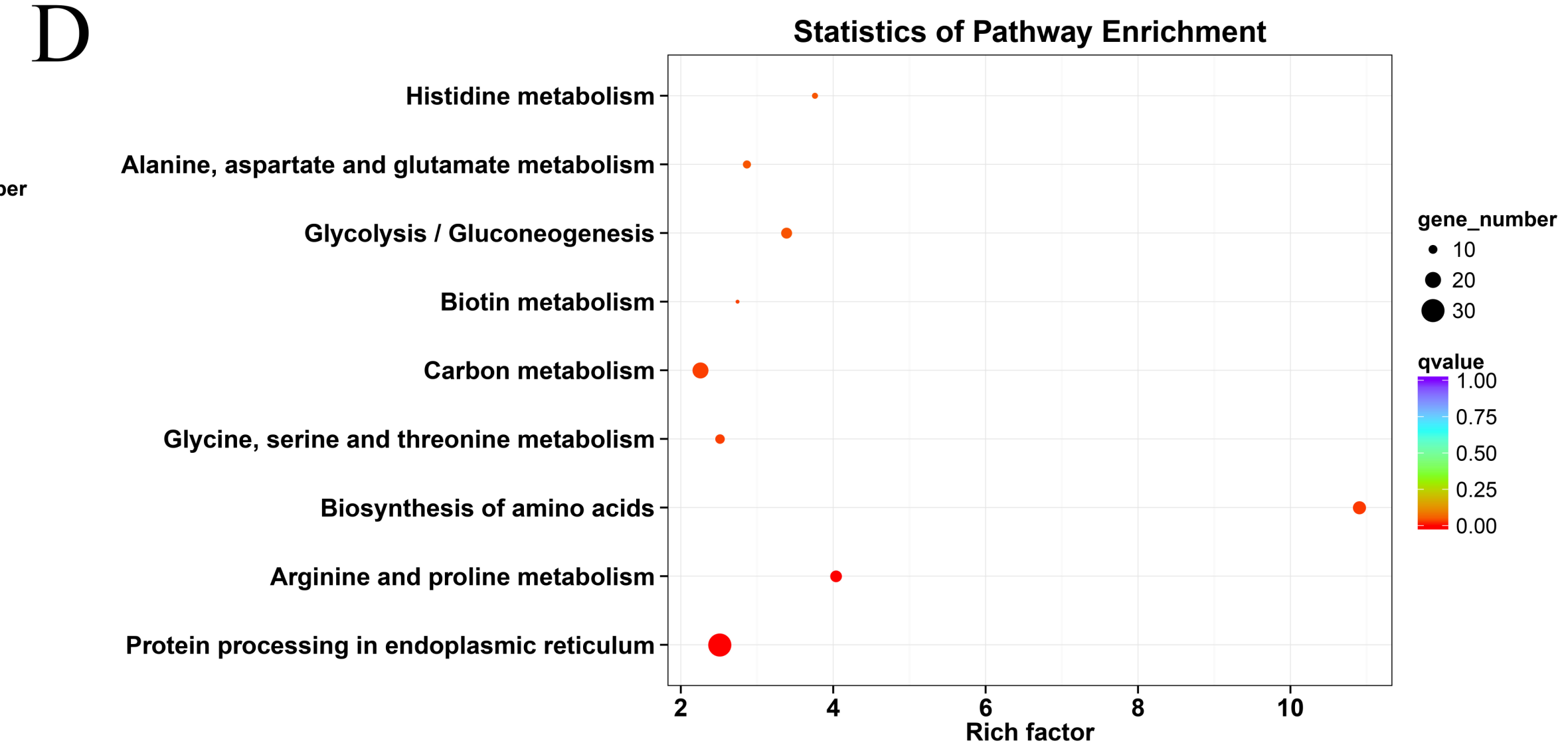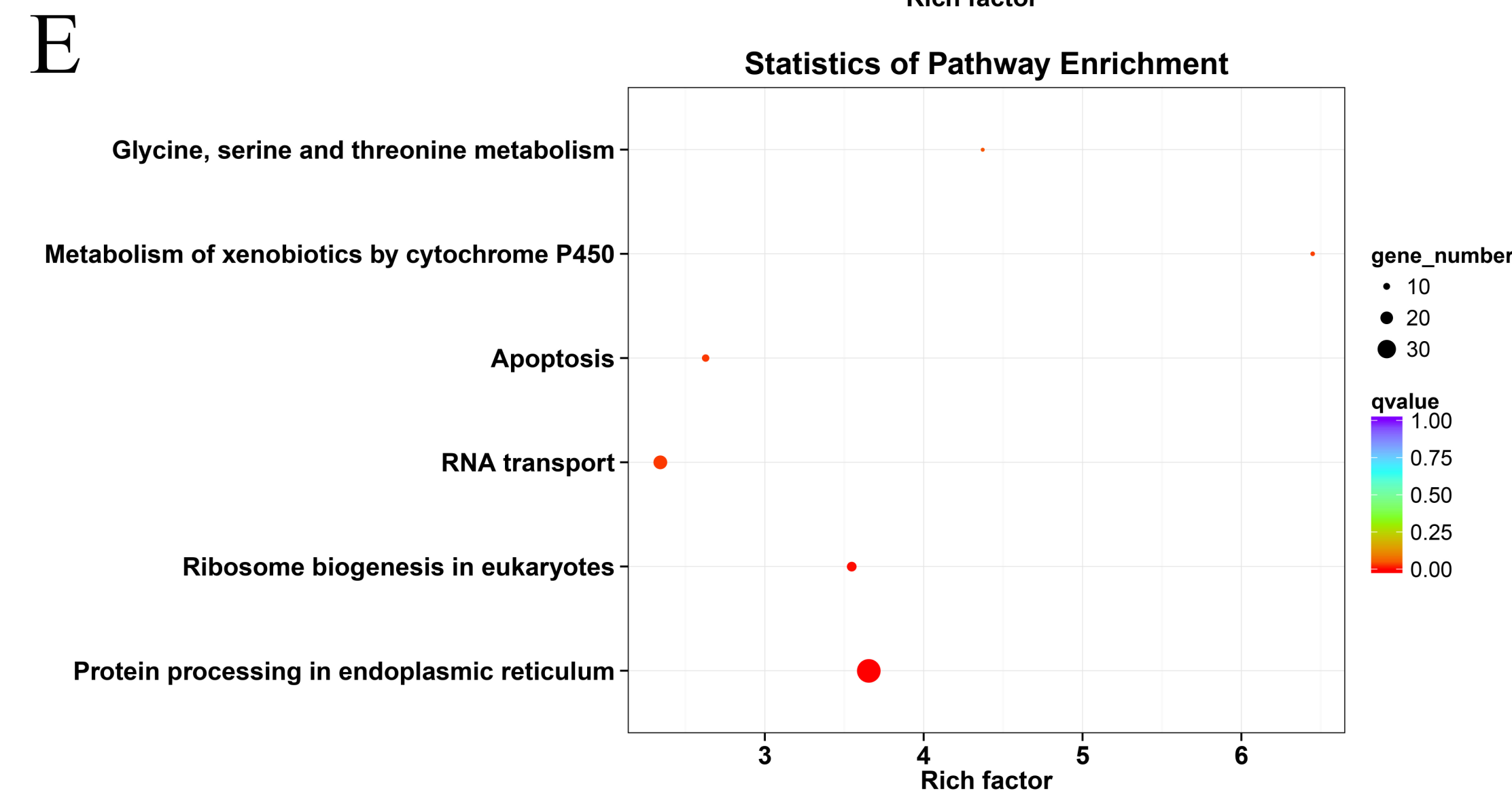

Supplement: Supplementary file 1 [file animals-11-03021-s001.zip › Files/Supplementary Figure S4.pdf]

A

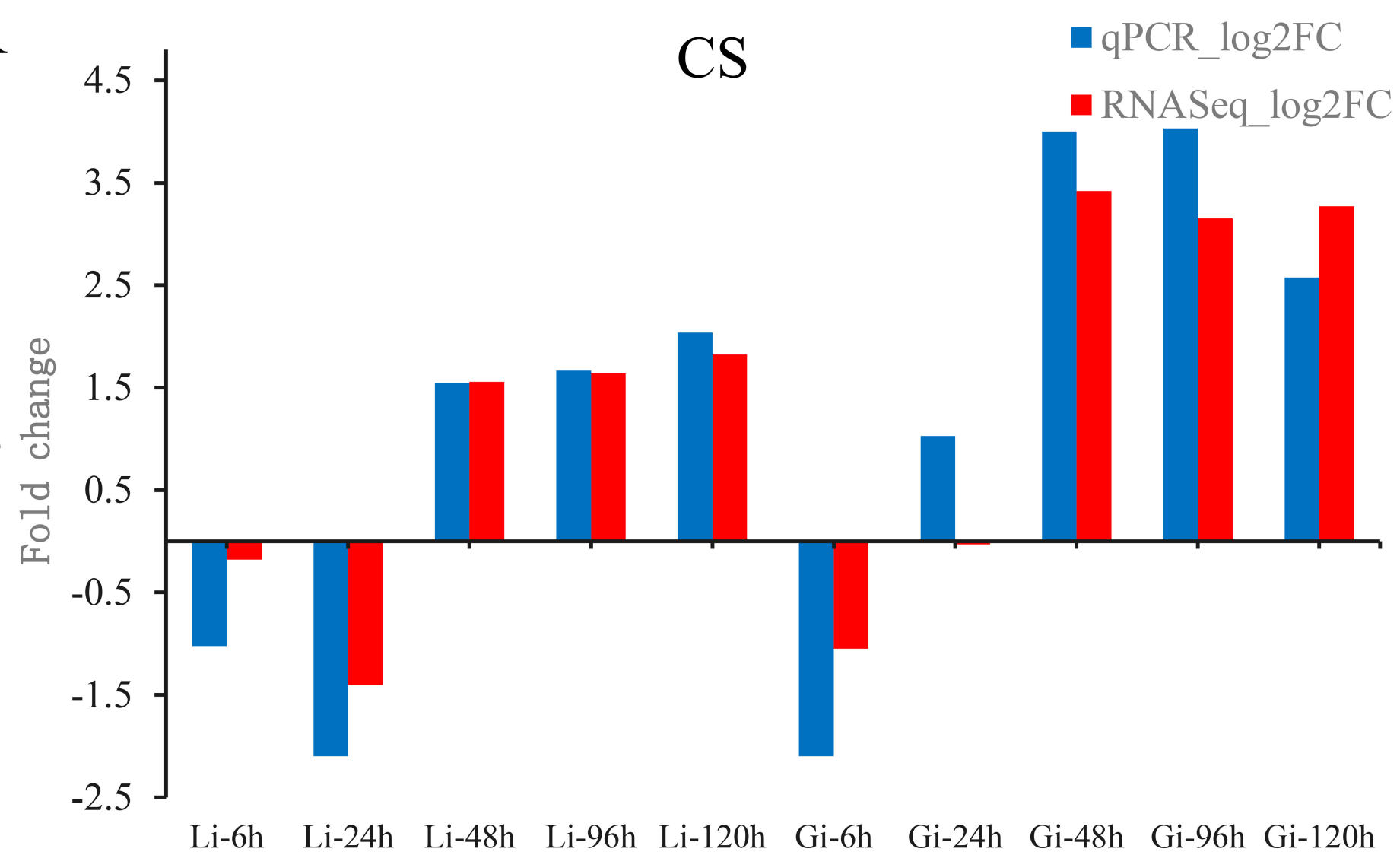

B

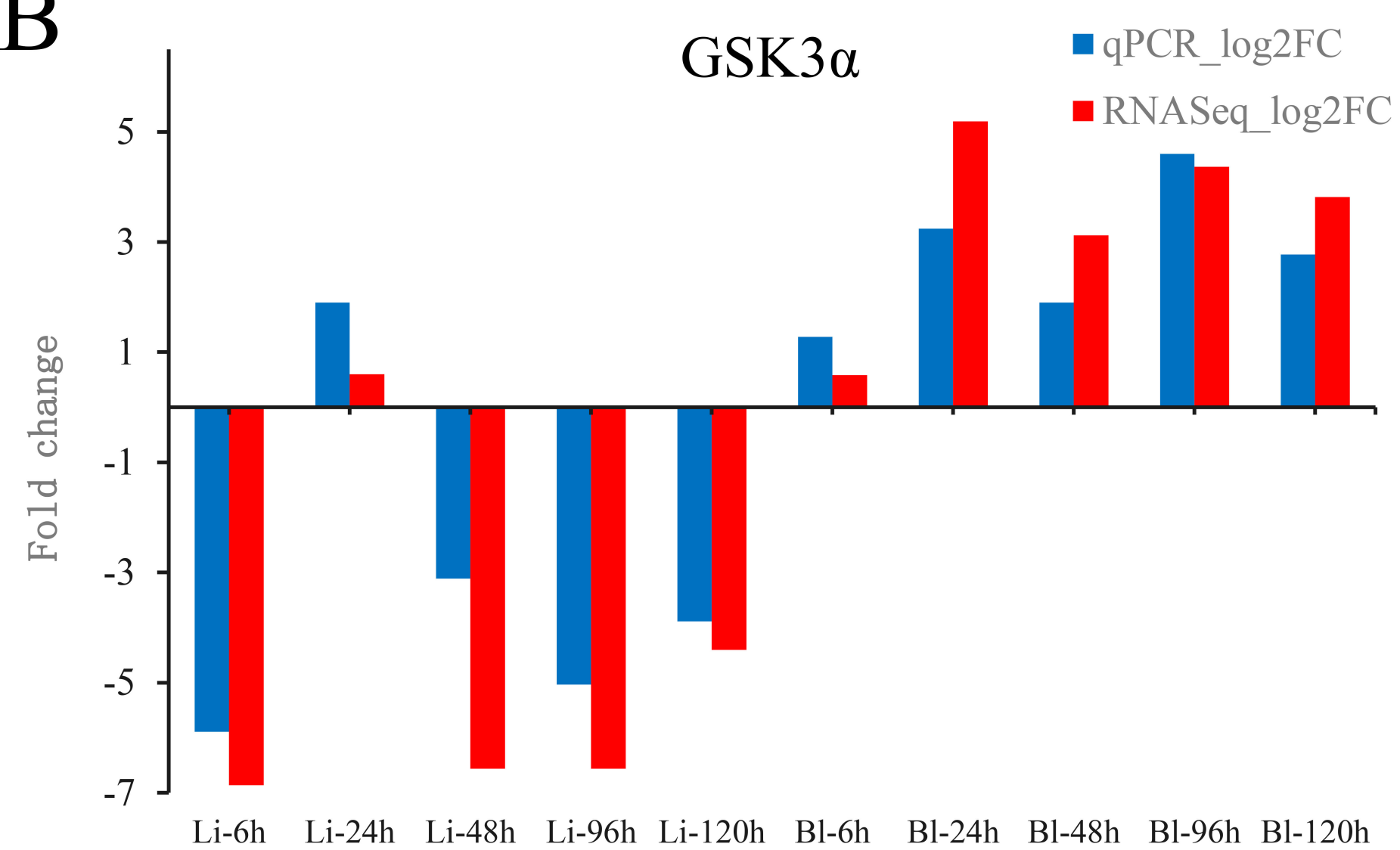

C

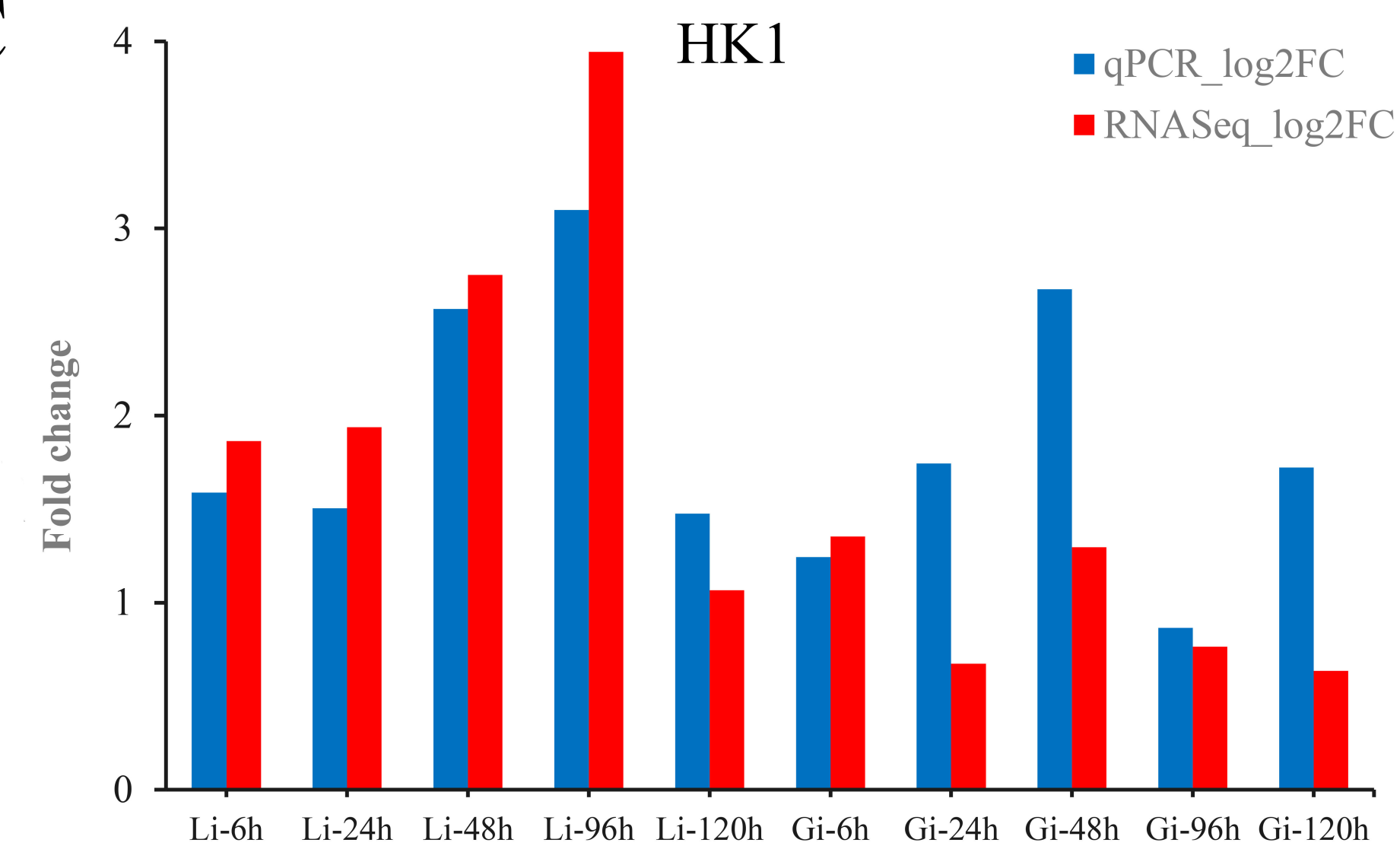

D

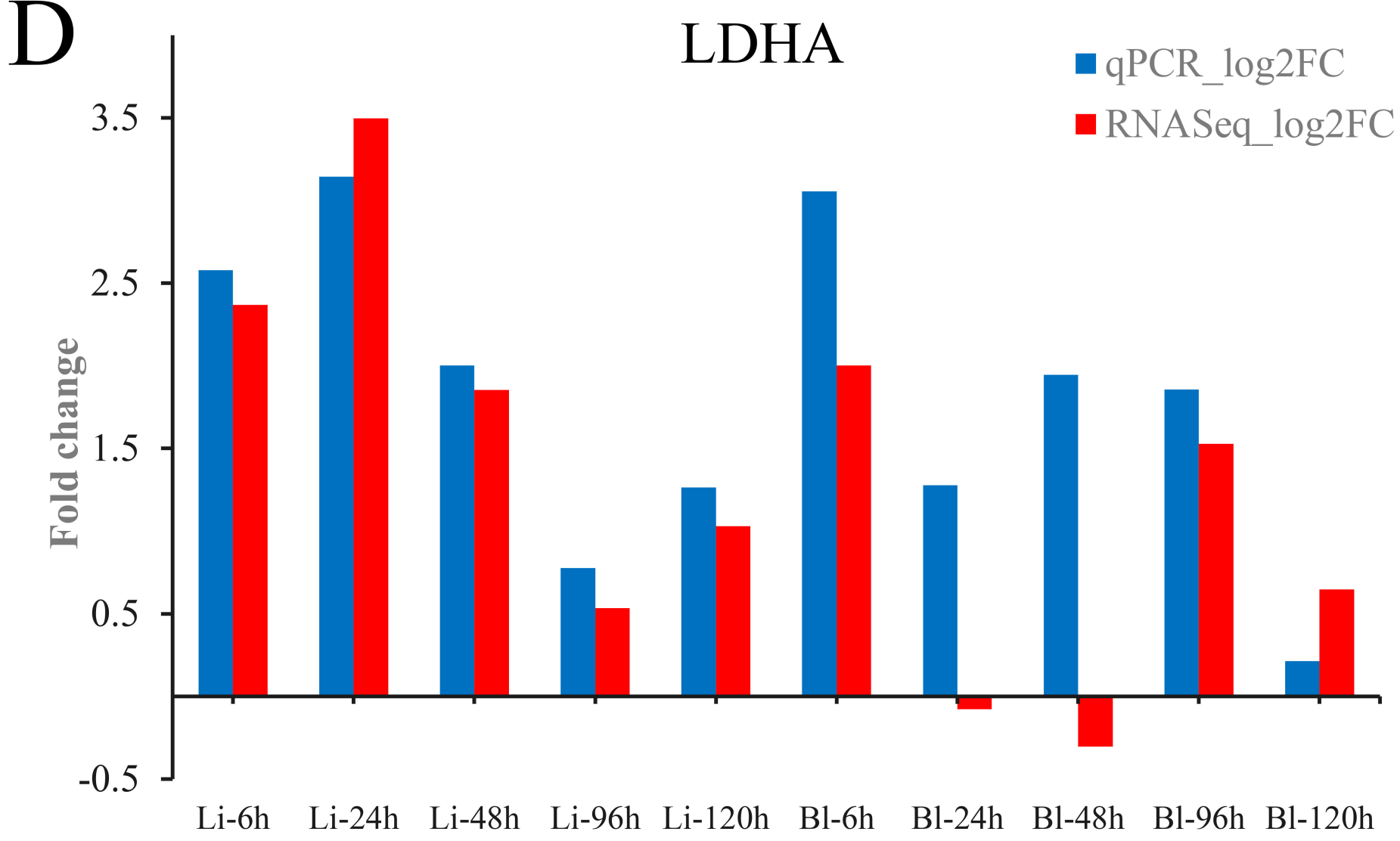

E

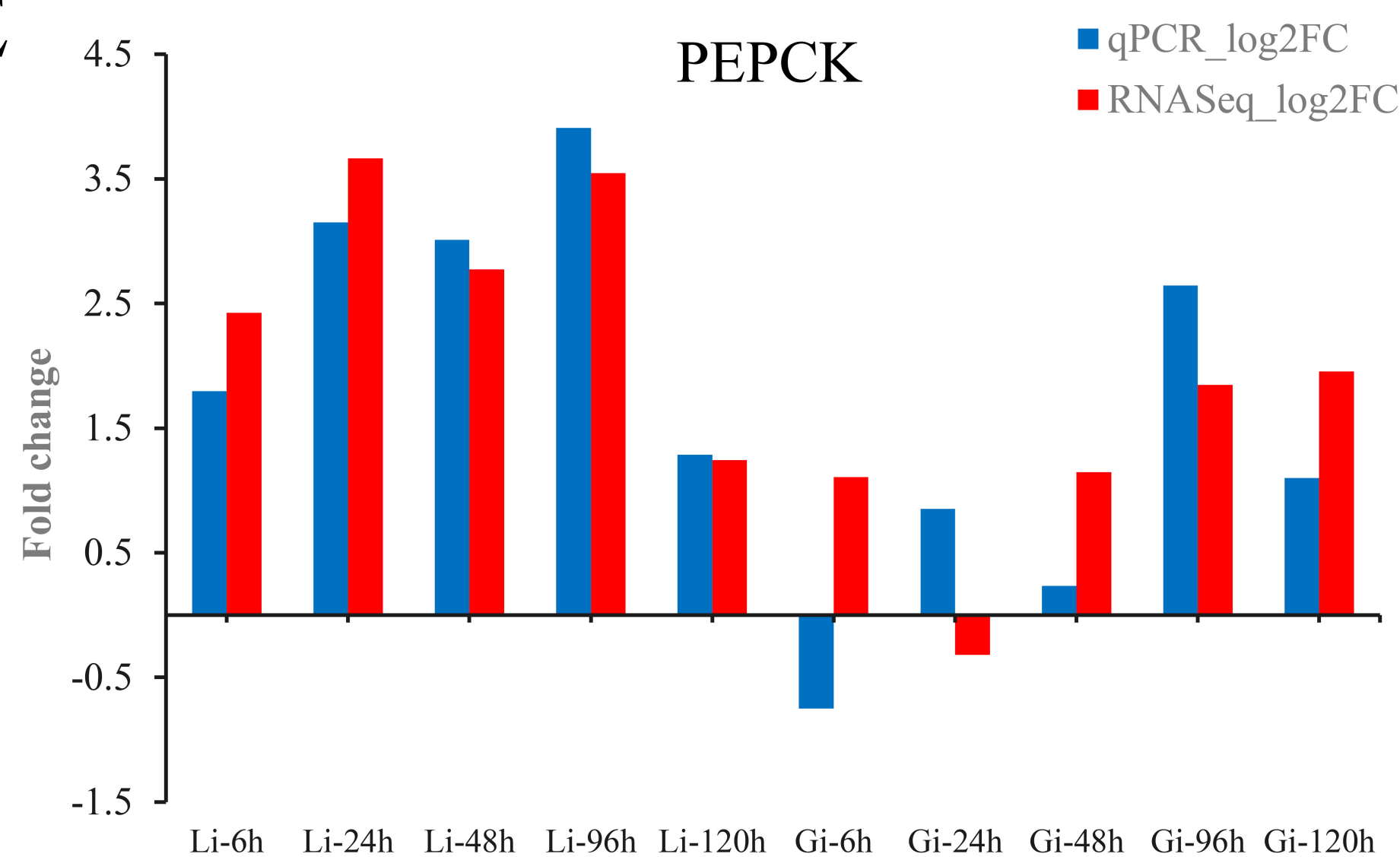

F

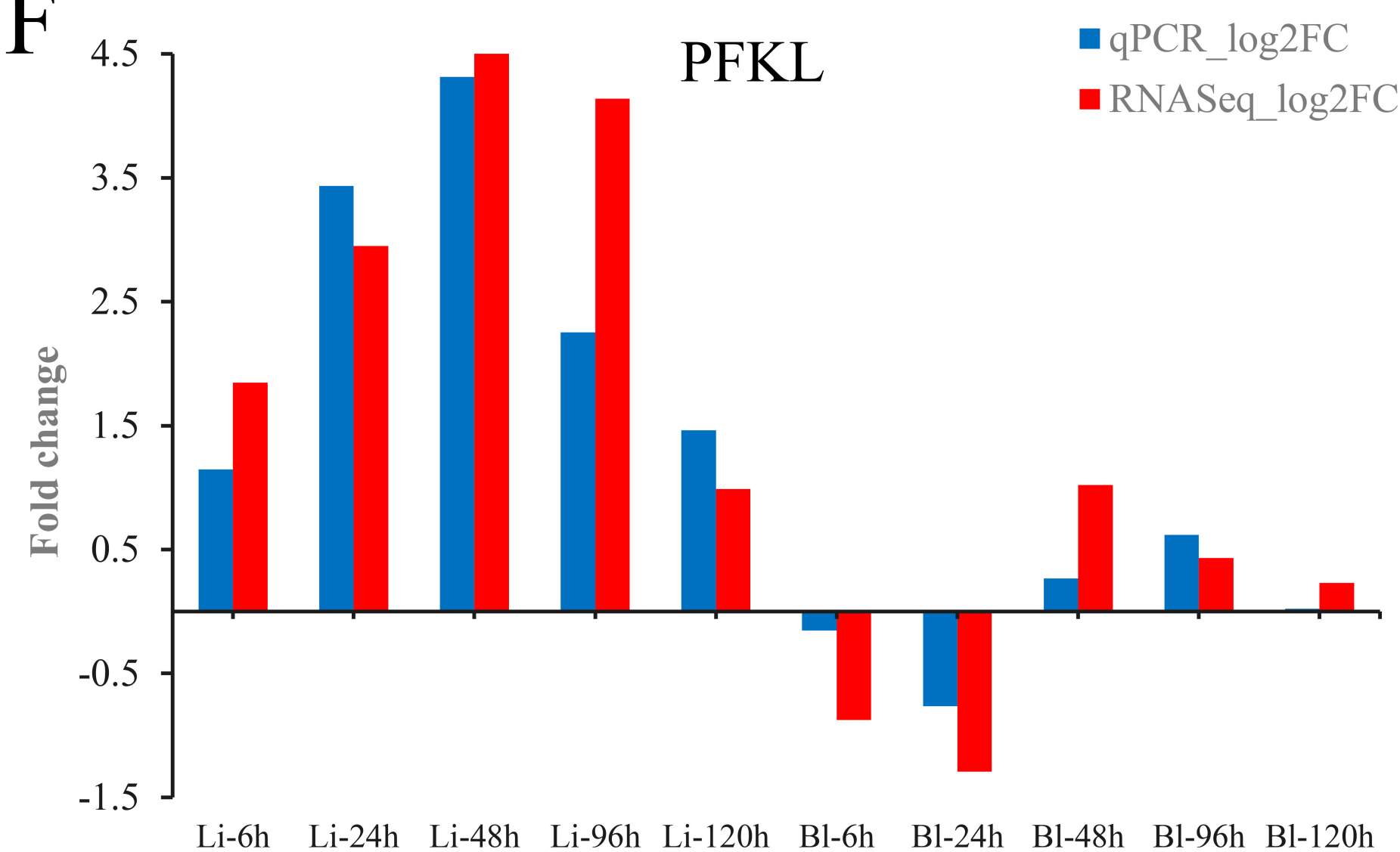

G

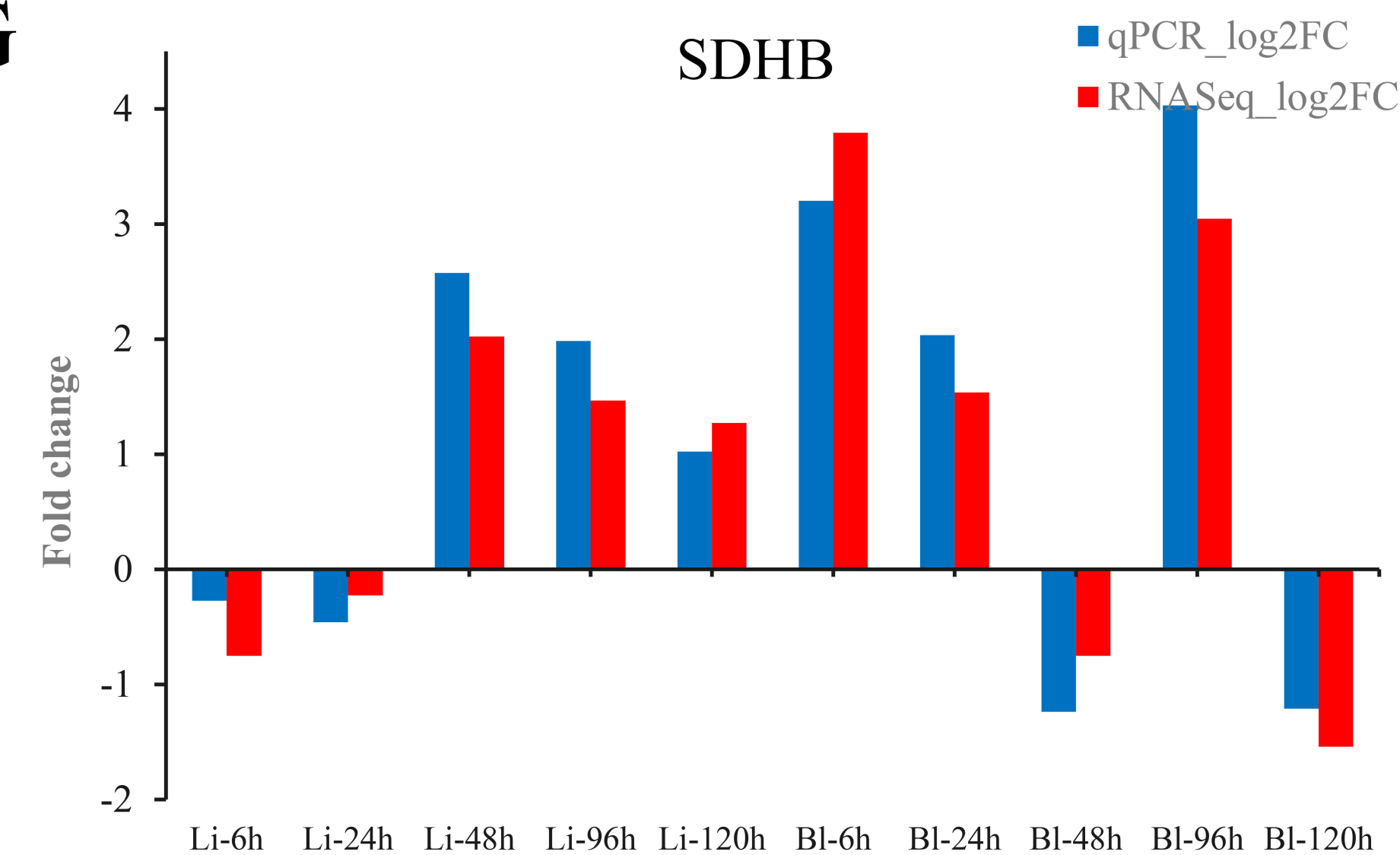

H

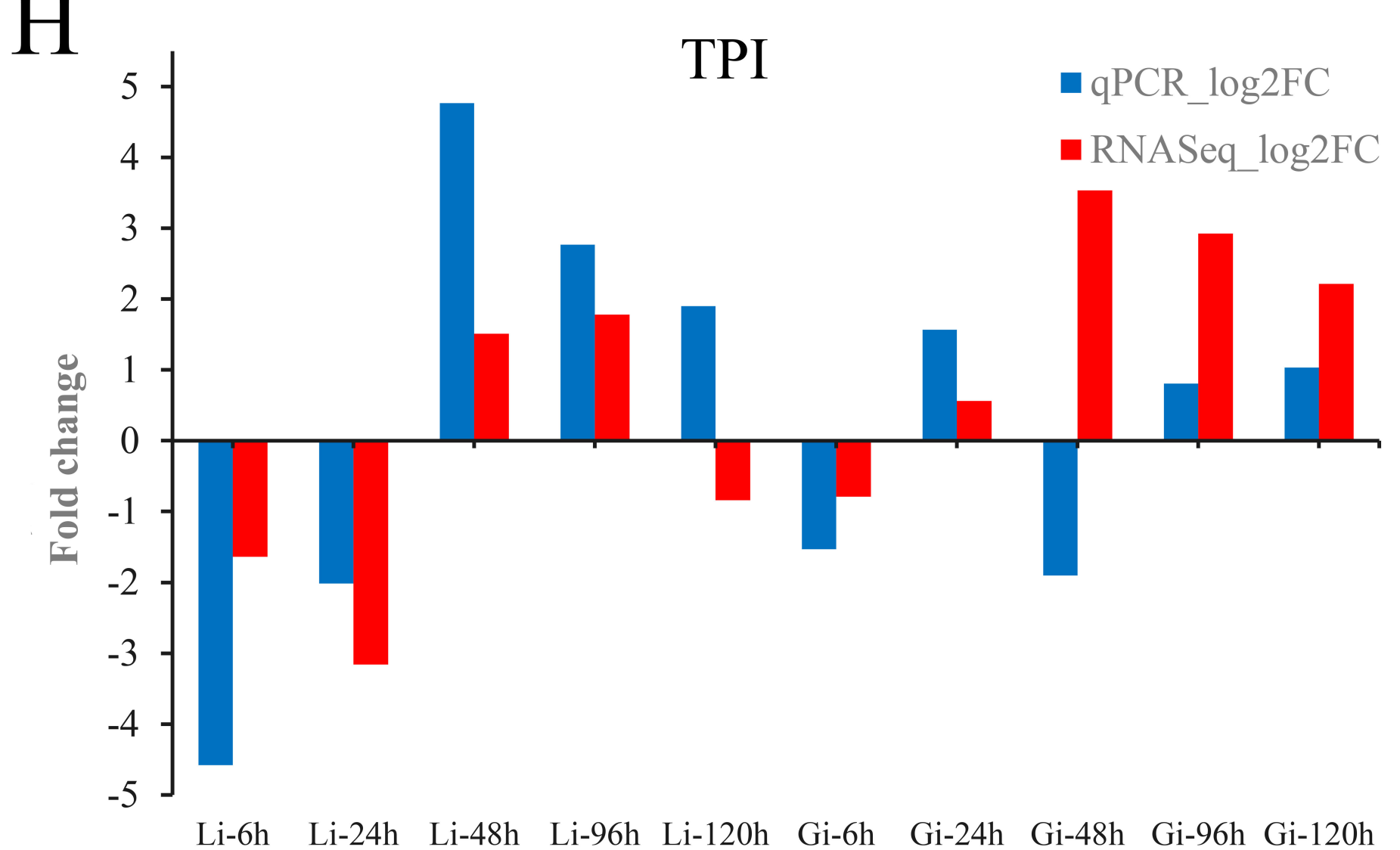

Supplement: Supplementary file 1 [file animals-11-03021-s001.zip › Files/Supplementary Figure S5.pdf]
